# SHMT2 Mediates Small‐Molecule‐Induced Alleviation of Alzheimer Pathology Via the 5′UTR‐dependent ADAM10 Translation Initiation

**DOI:** 10.1002/advs.202305260

**Published:** 2024-01-06

**Authors:** Li Song, Qiu‐Ling Pan, Gui‐Feng Zhou, Sheng‐Wei Liu, Bing‐Lin Zhu, Pei‐Jia Lin, Xiao‐Tong Hu, Jing‐Si Zha, Yan Long, Biao Luo, Jian Chen, Ying Tang, Jing Tang, Xiao‐Jiao Xiang, Xiao‐Yong Xie, Xiao‐Juan Deng, Guo‐Jun Chen

**Affiliations:** ^1^ Department of Neurology Chongqing Key Laboratory of Major Neurological and Mental Disorders The First Affiliated Hospital of Chongqing Medical University Chongqing 400016 China; ^2^ Department of Pharmacy Yongchuan Hospital of Chongqing Medical University Chongqing 402160 China; ^3^ Department of Health Management Daping Hospital Army Medical university Chongqing 400042 China; ^4^ Department of Internal Medicine The Southwest University Hospital Chongqing 400715 China; ^5^ Department of Geriatric Medicine Daping Hospital Army Medical university Chongqing 400042 China; ^6^ Department of Neurology West China Hospital Sichuan University Chengdu 610041 China; ^7^ Department of Nuclear Medicine The Second Affiliated Hospital of Chongqing Medical University Chongqing 400010 China

**Keywords:** 5′UTR, ADAM10, SHMT2, Alzheimer's disease, Kenpaullone, RNA binding protein, translation inhibitory element

## Abstract

It is long been suggested that one‐carbon metabolism (OCM) is associated with Alzheimer's disease (AD), whereas the potential mechanisms remain poorly understood. Taking advantage of chemical biology, that mitochondrial serine hydroxymethyltransferase (SHMT2) directly regulated the translation of ADAM metallopeptidase domain 10 (ADAM10), a therapeutic target for AD is reported. That the small‐molecule kenpaullone (KEN) promoted ADAM10 translation via the 5′ untranslated region (5′UTR) and improved cognitive functions in APP/PS1 mice is found. SHMT2, which is identified as a target gene of KEN and the 5′UTR‐interacting RNA binding protein (RBP), mediated KEN‐induced ADAM10 translation in vitro and in vivo. SHMT2 controls AD signaling pathways through binding to a large number of RNAs and enhances the 5′UTR activity of ADAM10 by direct interaction with GAGGG motif, whereas this motif affected ribosomal scanning of eukaryotic initiation factor 2 (eIF2) in the 5′UTR. Together, KEN exhibits therapeutic potential for AD by linking OCM with RNA processing, in which the metabolic enzyme SHMT2 “moonlighted” as RBP by binding to GAGGG motif and promoting the 5′UTR‐dependent ADAM10 translation initiation.

## Graphical abstract

SHMT2, an enzyme in mitochondrial one‐carbon metabolism, also functions as an RNA‐binding protein by interacting with the GAGGG motif in the 5’UTR of ADAM10. This interaction results in an altered 5’UTR structure that promotes ribosomal read‐through by the eIF2 complex. This mechanism enables the small molecule kenpaullone to enhance ADAM10 translation, thereby inhibiting Aβ generation associated with Alzheimer's disease.

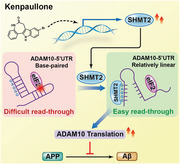

## 1 Introduction

Alzheimer's disease (AD) is pathologically characterized by amyloid β (Aβ) peptide deposition and neurofibrillary tangles in the brain.^[^ ^1^ ^]^ Whereas multiple mechanisms have been demonstrated,^[^ ^2^ ^]^ a large body of evidence suggests that one‐carbon metabolism (OCM) is closely associated with aging, the greatest risk factor for sporadic AD.^[^ ^3^ ^]^ Abnormal levels of metabolites and enzymes in OCM are found in the plasma and the brain of AD patients.^[^ ^4^ ^]^ Along with aging, deregulated metabolic pathways and OCM constitute the major molecular alterations in the hippocampus of APP/PS1 mice.^[^ ^5^ ^]^ However, how OCM might contribute to the pathogenesis of AD is incompletely understood.

Mitochondrial serine hydroxymethyltransferase (SHMT2) catalyzes the transfer of one‐carbon from serine to tetrahydrofolate (THF), leading to the production of glycine and 5,10‐methylene THF.^[^ ^6^ ^]^ SHMT2 is also required for proper mitochondrial translation initiation implicated in energy metabolism and ferroptosis.^[^ ^7^ ^]^ Deficiency of SHMT2 is embryonic lethal, and mutations of SHMT2 cause congenital microcephaly and intellectual disability.^[^ ^8^ ^]^ As SHMT2 expression is decreased with aging and in the entorhinal cortex of AD patients,^[^ ^9^ ^]^ it would be important to clarify whether SHMT2 might be involved in the pathophysiology of AD.

The α‐secretase ADAM metallopeptidase domain 10 (ADAM10) cleaves amyloid precursor protein (APP) and favors the non‐amyloid pathway, leading to a reduced level of Aβ.^[^ ^10^ ^]^ Growing evidence indicates that ADAM10 is a top candidate gene in late‐onset AD.^[^ ^11^ ^]^ Missense mutations in the prodomain of ADAM10 cosegregate with late‐onset AD, and a nonsense mutation of ADAM10 has been found in familial AD.^[^ ^12^ ^]^ Moreover, ADAM10 mRNA level is significantly decreased in the frontal cortex of AD patients.^[^ ^13^ ^]^ In specific regions of the healthy brain vulnerable to AD, the protein level of ADAM10 is reduced.^[^ ^14^ ^]^ Thus, ADAM10 could serve as a target for lead compound searching.^[^ ^15^ ^]^

Using luciferase‐based high‐throughput screenings, we have previously reported that some small molecules regulate amyloidogenesis,^[^ ^16^ ^]^ which leads to the finding of kenpaullone (KEN) in the present study. We define that KEN promotes the 5′ untranslated region (5′UTR)‐dependent ADAM10 translation and alleviates amyloidogenesis in APP/PS1 mice, an animal model of AD.^[^ ^17^ ^]^ SHMT2, which is identified as a target gene of KEN and the 5′UTR‐interacting RNA binding protein (RBP) as well, mediates the effect of KEN on ADAM10 and animal behaviors. Through binding to GAGGG motif that functions as a translation inhibitory element (TIE) and affects eIF2‐mediated read‐through processing, SHMT2 promotes the 5′UTR activity and ADAM10 translation, highlighting a novel mechanism in the regulation of the pathogenesis of AD.

## 2 Results

### 2.1 KEN Increased ADAM10 Expression in Human and Murine Cells

**Figure 1:**
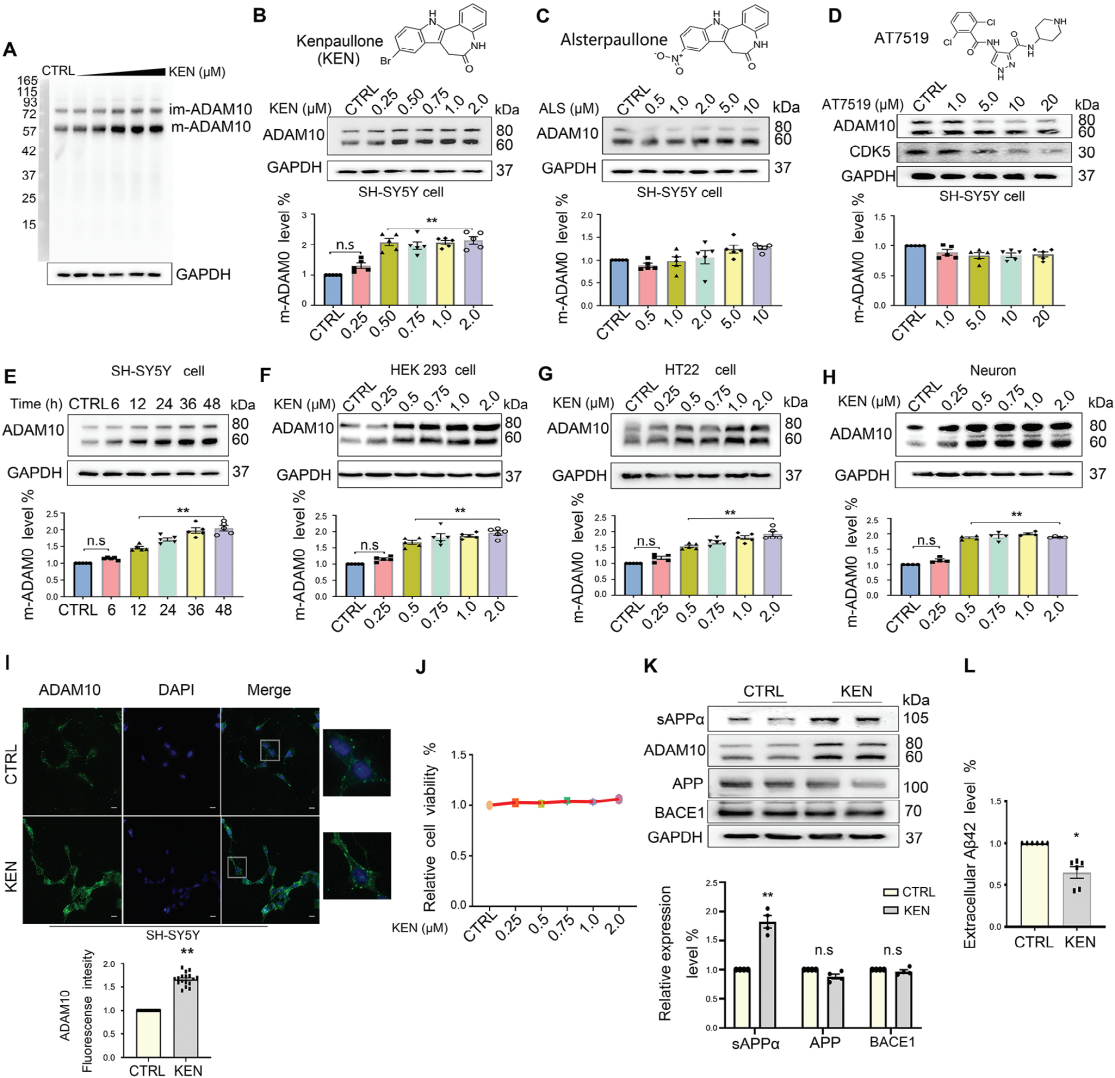
Kenpaullone (KEN) increases ADAM10 protein levels in human and murine cells. A) Full‐membrane immunoblots of ADAM10 show the immature (im‐ADAM10) and mature (m‐ADAM10) forms at a molecular weight of ≈80 and 60 kDa, respectively, in SH‐SY5Y cells treated with increased concentration of Kenpaullone (KEN). B) Chemical structure of KEN (top), immunoblots (middle), and quantification (bottom) of ADAM10 in SH‐SY5Y cells treated with KEN (0.25–2 µM for 36 h, *n* = 5). C) Chemical structure of KEN analog alsterpaullone (ALS, top), and immunoblots (middle) and quantification (bottom) of ADAM10 in SH‐SY5Y cells treated with ALS (0.5–10 µM for 36 h, *n* = 5). D) Chemical structure of another CDK/GSK inhibitor AT7519 (top), and immunoblots (middle) and quantification (bottom) of ADAM10 in SH‐SY5Y cells treated with AT7519 (1 to 20 µM for 36 h, *n* = 5). E) Time‐course effect of KEN (0.75 µM) on ADAM10 protein levels in SH‐SY5Y cells (*n* = 5). (F‐H) Immunoblots (top) and quantification (bottom) of ADAM10 treated with KEN (0.25–2 µM for 36 h) in human HEK293 cell line F), murine hippocampal cell line G) and primary cortical neurons (H), respectively (*n* = 5). (I) Immunofluorescent images (top) and quantification (bottom) of ADAM10 signals (green) in SH‐SY5Y cells treated with KEN (0.75 µM for 36 h). DAPI is a nuclear marker (blue), scale bar = 25 µm (*n* > 10). **(J)** Cell viability of SH‐SY5Y cells treated with KEN (0.25 to 2 µM for 36 h) measured by CCK‐8 kit (*n* = 5). **(K)** Immunoblots (top) and quantification (bottom) of sAPPα, ADAM10, APP, and BACE1 in SH‐SY5Y‐APP cells treated with KEN (0.75 µM for 36 h, *n* = 4). (L) Aβ1‐42 levels in conditioned medium of SH‐SY5Y‐APP cells treated with KEN (0.75 µM for 36 h, *n* = 6). All values were normalized to vehicle control (CTRL) in each experiment. Data are presented as mean ± SEM from three or more independent experiments. n.s: no significant difference. ^*^ *p* < 0.05, ^**^ *p* < 0.01.

It is reported that KEN modulates parkin recruitment to mitochondria,^[^ ^18^ ^]^ prevents hearing loss,^[^ ^19^ ^]^ increases neuronal differentiation,^[^ ^20^ ^]^ and prolongs healthy survival of motor neurons.^[^ ^21^ ^]^ Intriguingly, this molecule seems to have anticancer activity but is also anti‐apoptotic in neurodegeneration.^[^ ^22^ ^]^ To explore the potential role of KEN in ADAM10 expression, we first measured ADAM10 protein levels in SH‐SY5Y (neuroblastoma) cells. As shown in **Figure** **1**A, full‐membrane immunoblots of ADAM10 showed the immature (im‐ADAM10) and mature (m‐ADAM10) forms at a molecular weight of ≈80 and 60 kDa, respectively.^[^ ^23^ ^]^ As m‐ADAM10 is the functional form that possesses the α‐secretase activity in amyloidogenesis, this form was chosen for further analysis. Compared with vehicle control, KEN significantly increased the expression of m‐ADAM10 at concentrations ranging from 0.5 to 2 µM (Figure 1B). To determine whether this effect was attributed to the inhibition of CDKs and GSK‐3 by KEN,^[^ ^24^ ^]^ we assessed ADAM10 protein level in cells treated with the KEN analog alsterpaullone (ALS) and AT7519 that has a different structure.^[^ ^25^ ^]^ Surprisingly, whereas a slight but not significant increase of m‐ADAM10 protein levels was found in ALS‐treated cells (0.5 to 10 µM, Figure 1C), no significant alteration of m‐ADAM10 was detected in cells incubated with AT7519 (1 to 20 µM, Figure 1D), suggesting that enhancement of ADAM10 was dependent on the unique structure of KEN rather than kinase activity. The time‐course effect showed that elevation of ADAM10 levels by KEN started at 12 h and lasted for 48 h (Figure 1E). KEN also enhanced ADAM10 protein levels in human HEK293 cells, murine HT22 (hippocampal) cells, and primary cortical neurons (Figure 1F–H). In support of Western blotting results, immunofluorescent images also showed that ADAM10 protein expression was significantly increased in SH‐SY5Y cells by KEN, which was without noticeable cellular toxicity (Figure 1I,J). We further found that the level of soluble APP α (sAPPα), a catalytic product of APP by ADAM10, was significantly increased. In comparison, that of APP and BACE1 was not significantly altered (Figure 1K). As expected, Aβ42 levels were significantly decreased in KEN‐treated SH‐SY5Y cells that stably express human full‐length APP 695 (SH‐SY5Y‐APP) (Figure 1L), suggesting that the elevated ADAM10 by KEN was functional in Aβ generation. These results indicated that KEN enhanced ADAM10 in human and murine cells, including neurons.

### 2.2 KEN‐Mediated Enhancement of ADAM10 was Dependent on the 5′UTR

**Figure 2:**
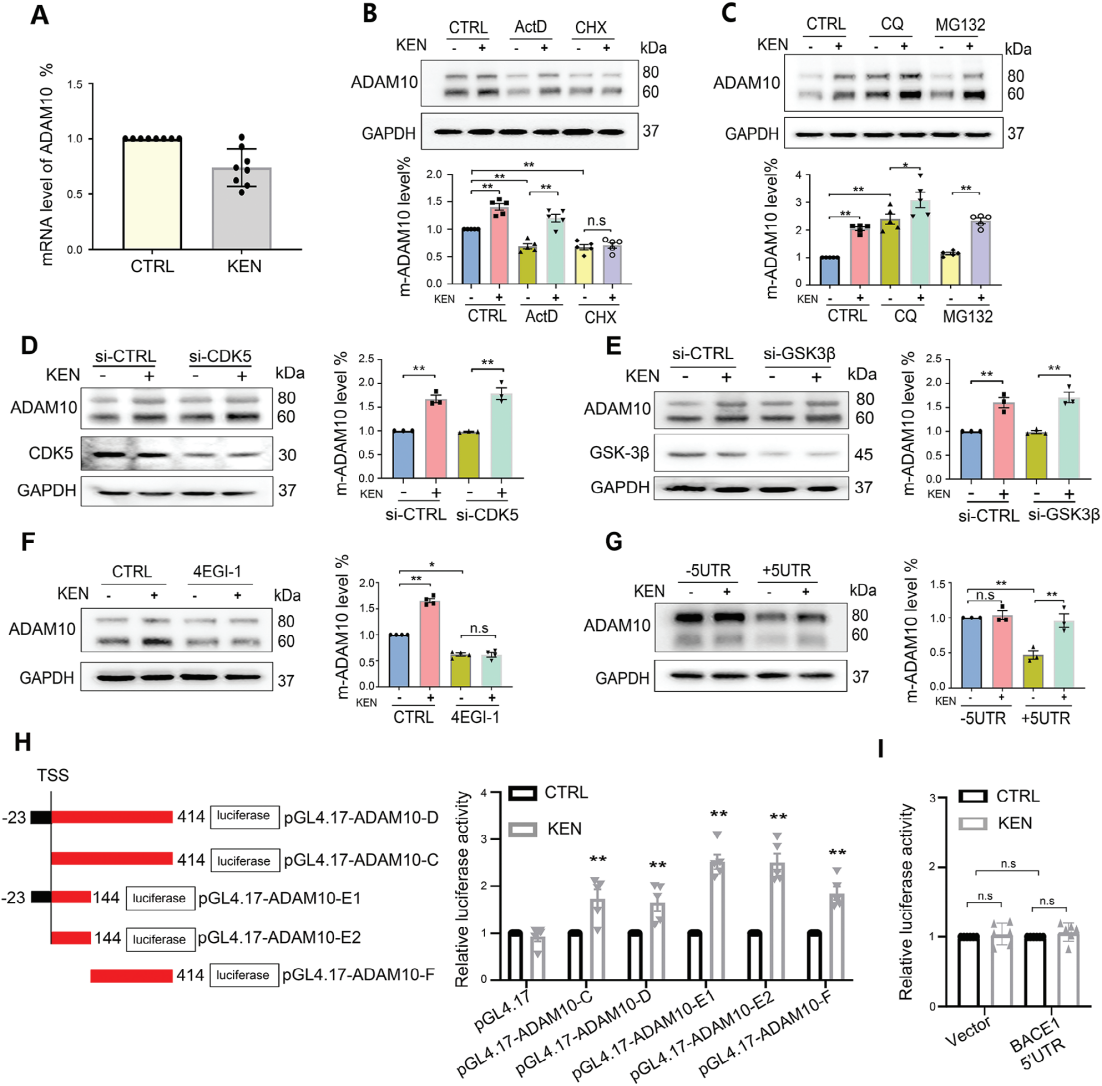
KEN‐induced ADAM10 translation is dependent on the 5′UTR. A) Relative mRNA levels of ADAM10 in SH‐SY5Y cells treated with KEN (0.75 µM for 36 h, *n* = 8). B) Immunoblots (top) and quantification (bottom) of ADAM10 in SH‐SY5Y cells treated with KEN (0.75 µM for 36 h), in the absence or presence of transcription inhibitor actinomycin D (ActD, 0.1 µM for 12 h) or protein synthesis inhibitor cycloheximide (CHX, 10 µM for 8 h), respectively (*n* = 5). C) Immunoblots (top) and quantification (bottom) of ADAM10 in SH‐SY5Y cells treated with KEN (0.75 µM for 36 h), in the absence or presence of lysosome inhibitor chloroquine (CQ, 100 µM for 6 h) or proteasome inhibitor MG132 (1 µM for 6 h), respectively (*n* = 5). D) Immunoblots (left) and quantification (right) in SH‐SY5Y cells incubated with KEN (0.75 µM for 36 h) and transiently transfected with CDK5 siRNA (siCDK5) for 48 h (*n* = 3). E) Immunoblots (left) and quantification (right) in SH‐SY5Y cells incubated with KEN (0.75 µM for 36 h) and transiently transfected with GSK‐3β siRNA (siGSK‐3β) for 48 h (*n* = 3). F) Immunoblots (left) and quantification (right) of ADAM10 in SH‐SY5Y cells treated with KEN (0.75 µM for 36 h), in the absence or presence of a competitive inhibitor of eIF4E/eIF4G (4EGI1, 50 µM for 24 h, *n* = 4). G) Immunoblots (left) and quantification (right) of ADAM10 in HEK293 cells transiently transfected with human ADAM10 constructs in which the 5′UTR sequence was deleted (−5′UTR) or included (+ 5′UTR), in the absence (CTRL) or presence of KEN (0.75 µM for 36 h, *n* = 3). H) Left: the schematic diagram shows that the 5′UTR of ADAM10 is truncated into different fragments and subcloned into a pGL4.17 vector to construct the luciferase reporter plasmids: pGL4.17‐ADAM10‐D, C, E1/E2 and F, respectively. Numbers indicate the relative positions with respect to nucleotides in the 5′UTR. Right: relative luciferase activities in SH‐SY5Y cells transiently transfected with different pGL4.17‐ADAM10 plasmids in the absence (CTRL) or presence of KEN (0.75 µM for 36 h). The tested luciferase activity in each group was normalized to the value of the internal control plasmid pGL4.17. I) Relative luciferase activities in SH‐SY5Y cells transiently transfected with pmirGLO/pGL4.51 plasmid that was without (vector) or with BACE1 5′UTR for 12 h, in the absence (CTRL) or presence of the following KEN (0.75 µM for 36 h) treatment. Data are presented as mean ± SEM from three or more independent experiments. All values were normalized to vehicle control (CTRL) in each experiment. n.s: no significant difference. ^*^ *p* < 0.05, ^**^ *p* < 0.01.

The elevated ADAM10 could be a result of the altered transcription, translation, or protein degradation, thus we first measured the mRNA levels in SH‐SY5Y cells. As shown in **Figure** **2**A, ADAM10 mRNA was not altered by KEN. Transcription inhibitor actinomycin D (ActD) or protein synthesis inhibitor cycloheximide (CHX) alone led to a reduced ADAM10 protein level,^[^ ^26^ ^]^ whereas KEN‐induced ADAM10 enhancement was diminished in the presence of CHX but not ActD (Figure 2B), suggesting an involvement of protein synthesis. It seemed that protein degradation machinery was not involved in this regulation, as the proteasomal inhibitor MG132 or the lysosomal inhibitor chloroquine (CQ)^[^ ^16a^ ^]^ failed to block KEN‐mediated augmentation of ADAM10 (Figure 2C). Moreover, we excluded the possibility that CDKs/GSK‐3 played a role in ADAM10 regulation, as silencing of CDK5 or GSK‐3β alone did not change ADAM10 protein under basal condition and failed to further prevent KEN‐induced enhancement of ADAM10 protein levels (Figure 2D,E). Thus, we next assessed whether translation machinery mediates the effect of KEN. As shown in Figure 2F, the translation inhibitor 4EGI1 that disrupts the eukaryotic translation initiation factors E (eIF4E)‐eIF4G interaction^[^ ^27^ ^]^ significantly reduced the basal ADAM10 protein level and further attenuated the enhancement of ADAM10 by KEN. Using transiently introduced ADAM10 constructs with the 5′UTR either included (+5′UTR) or deleted (−5′UTR), we found that the absence of 5′UTR significantly enhanced the basal level of ADAM10 as previously reported,^[^ ^28^ ^]^ and KEN‐induced enhancement of ADAM10 was diminished in −5′UTR but not +5′UTR (Figure 2G). Further 5′UTR‐luciferase assay showed that nucleotides 1–144 and 145–414 were sufficient to mediate the function of KEN (Figure 2H), whereas the 5′UTR activity of BACE1 was not altered (Figure 2I), suggesting a selective regulation of ADAM10. These results indicated that KEN induced ADAM10 translation via the 5′UTR.

### 2.3 KEN Promoted ADAM10 Expression and Rescued Cognitive Deficits in APP/PS1 Mice

**Figure 3:**
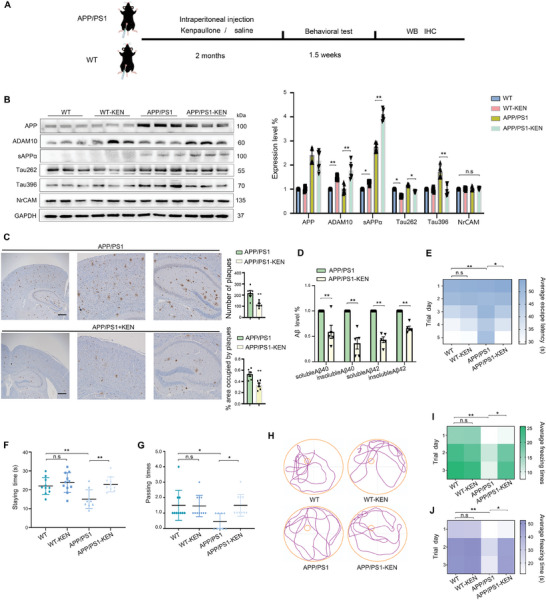
KEN enhances ADAM10 and rescues cognitive deficits in APP/PS1 mice. A) Schematic diagram represents the time course of the in vivo test of KEN. B) Immunoblots (left) and quantification (right) of APP, sAPPα, ADAM10, Tau396, Tau262, and NrCAM in the hippocampus. Wild‐type (WT) and APP/PS1 mice (female at 12 months) were administered intraperitoneally with vehicle (CRTL) or KEN (7 mg/Kg, every other day) for 2 months, leading to four groups: WT, WT‐KEN, APP/PS1 and APP/PS1‐KEN, respectively (*n* = 6). C) Immunohistochemical images (left) and quantification (right) of Aβ deposition in the brain of APP/PS1 and APP/PS1‐KEN, scale bar = 500 µm (*n* = 6). D) The soluble and insoluble Aβ1‐40/1‐42 levels in the hippocampus of APP/PS1 and APP/PS1‐KEN, respectively (*n* = 5). E–J) Spatial and associative learning and memory performances are assessed by Morris water maze tests E–H) followed by fear conditioning tests (I&J), respectively. E) The heatmap shows in the hidden platform tests, the time (seconds, s) spent in searching for the platform (average escape latency) in different trial days (1–5), is compared among WT, WT‐KEN, APP/PS1 and APP/PS1‐KEN, respectively. *n* = 9 to 11. F,G) To seek the site where the hidden platform was previously located, the time period of staying (F, staying time) and the number of times crossed the site (G, passing times) are compared. H) Representative movement trajectories of mice in different groups on the sixth day. I,J) The heatmaps show the number of freezing (I, freezing times) and duration of freezing (J, freezing time in seconds) in different groups, and on different trial days (1–3). *n* = 6–8. All values were normalized to vehicle control (CTRL) in each experiment. Data are presented as mean ± SEM from three or more independent experiments. n.s: no significant difference. ^*^ *p* < 0.05, ^**^ *p* < 0.01.

We next determined whether the enhanced ADAM10 expression correlates with the altered amyloidogenesis and cognitive functions in vivo (**Figure** **3**A). Wild‐type (WT) and APP/PS1 mice were administered intraperitoneally with vehicle (CRTL) or KEN, which led to the following four groups: WT, WT‐KEN, APP/PS1, and APP/PS1‐KEN, respectively. Given that KEN is an inhibitor of CDKs and GSK‐3 that are known to phosphorylate Tau at selective sites including Ser262 (Tau262) and Ser396 (Tau396),^[^ ^29^ ^]^ which are elevated in the brain of APP/PS1 mice.^[^ ^30^ ^]^ Thus, the effectiveness of KEN in the brain could be verified by measuring Tau262/396 level. Moreover, one of the substrates of ADAM10, neural glial‐related cell adhesion molecule (NrCAM) that controls neurite outgrowth, has been used to evaluate the side‐effects of ADAM10 activators.^[^ ^31^ ^]^ As shown in Figure 3B, the protein level of Tau262/396 was significantly decreased in APP/PS1‐KEN relative to APP/PS1, indicating that KEN successively reached the brain and exerted a biological function. As expected, the protein levels of ADAM10 and sAPPα, but not NrCAM, were significantly elevated in both WT and APP/PS1 mice (Figure 3B), indicating that KEN selectively enhanced ADAM10 while sparing neurite outgrowth associated side‐effects.^[^ ^31^ ^]^ We further showed that cerebral Aβ deposits (Figure 3C), and the levels of soluble and insoluble Aβ1‐40/1‐42 in the hippocampus were significantly reduced (Figure 3D). These results indicated that in KEN‐treated APP/PS1 mice, enhancement of ADAM10 protein levels was with the concomitant reduction of Aβ deposits.

To determine whether KEN may affect cognitive functions, we assessed spatial and associative learning memory using water maze tests and context fear conditioning tests in APP/PS1 mice.^[^ ^32^ ^]^ In the hidden platform test, the escape latency was significantly shorter in APP/PS1‐KEN than in APP/PS1 beginning on the third day (Figure 3E). When the platform was removed during the probe trial, the staying time in the target quadrant (Figure 3F) and the passing time for crossing over the target site (Figure 3G) were significantly longer in APP/PS1‐KEN than in APP/PS1, indicating an improved spatial memory by KEN (Figure 3H). The subsequent context fear conditioning tests revealed that the number of freezing (freezing times) was significantly larger and the duration of freezing (freezing time) significantly longer in APP/PS1‐KEN than in APP/PS1 (Figure 3I,J). No significant differences were observed between WT and WT‐KEN (Figure 3E–J). These results indicated that KEN significantly improved spatial and associative learning memory in APP/PS1 mice.

### 2.4 SHMT2 was a Target Gene of KEN and the 5′UTR‐Interacting RNA Binding Protein

**Figure 4:**
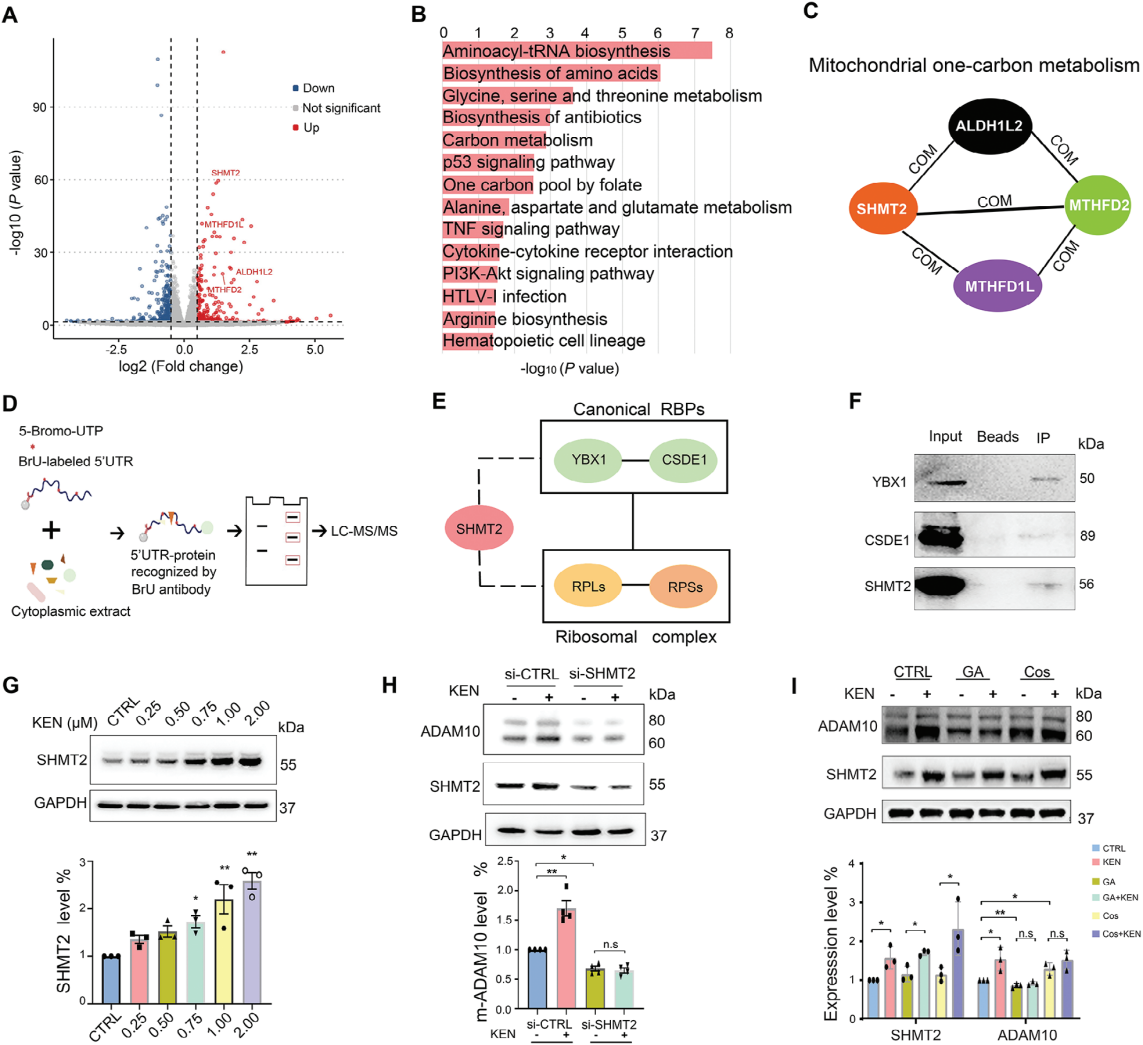
SHMT2 is a target gene of KEN and the 5′UTR‐interacting RBP. A) Volcano plots of differentially expressed genes in SH‐SY5Y‐APP cells induced by KEN (0.75 µM for 36 h). SHMT2, along with other mitochondrial genes associated with one‐carbon metabolism including ALDH1L2, MTHFD2, and MTHFD1L, is significantly increased by KEN. B) KEGG pathways were analyzed from the significantly up‐regulated genes by KEN. C) A simplified PPI network shows SHMT2 interacts with other mitochondrial genes upregulated by KEN. PPI‐network was built according to KEGG database and constructed by Cytoscape. Com: complex. D) Whole lysates of SH‐SY5Y cells treated with or without KEN (0.75 µM for 36 h) were mixed with BrU‐labeled 5′UTR and anti‐BrU conjugated beads, the RNA‐proteins recognized by BrU antibody were subjected to SDS/PAGE, and silver‐stained protein bands were used for LC/MS‐MS analysis. E) A simplified PPI‐network based on STRING online‐analysis shows that SHMT2 is associated with the classical RBPs including YBX1 and CSDE1, and ribosome complex proteins. F) Immunoblots of YBX1, CSDE1, and SHMT2 by the 5′UTR‐targeted RNA‐pulldown assay. G) Immunoblots (top) and quantification (bottom) of SHMT2 protein in SH‐SY5Y cells treated with KEN at indicated concentrations for 36 h (*n* = 3). (H) Immunoblots (top) and quantification (bottom) of ADAM10 in HEK293 cells incubated with KEN (0.75 µM for 36 h, *n* = 4) and transiently transfected with SHMT2 siRNA (siSHMT2) for 48 h. (I) Immunoblots (top) and quantification (bottom) of SHMT2 and ADAM10 in SH‐SY5Y cells treated with KEN (0.75 µM for 36 h), in the absence or presence of SHMT2 inhibitor Glycyrrhetinic acid (GA, 100 µM for 48 h) or the 5′UTR‐dependent ADAM10 enhancer Cosmosiin (Cos, 5 µM for 36 h), respectively (*n* = 3). All values were normalized to vehicle control (CTRL) in each experiment. Data are presented as mean ± SEM from three or more independent experiments. n.s: no significant difference. ^*^ *p* < 0.05, ^**^ *p* < 0.01.

To further understand the potential mechanisms in KEN‐induced ADAM10 translation, we assessed differentially regulated genes (DEGs) by RNA‐seq in SH‐SY5Y‐APP cells, in which Aβ is overproduced thus remodeling amyloidogenesis in AD‐like pathology.^[^ ^26^, ^33^ ^]^ Co‐expression networks were built to find relations among genes in the absence and presence of KEN (Figure S1, Supporting Information). KEN induced a total of 733 DEGs including 296 upregulated and 437 downregulated genes (**Figure** **4**A and Supplemental Table‐DEGs). The upregulated pathways (red) included aminoacyl‐tRNA biosynthesis, biosynthesis of amino acids, carbon metabolism, and one carbon pool by folate (Figure 4B). Importantly, SHMT2 was identified as a hub gene in mitochondrial OCM, which included aldehyde dehydrogenase one family member L2 (ALDH1L2), methylenetetrahydrofolate dehydrogenase (NADP+ dependent) 2 (MTHFD2) and MTHFD1L (Figure 4C, Figure S2, Supporting Information).

The 5′UTR dependence prompted us to speculate that some of DEGs might also function as RBPs in the regulation of ADAM10 translation. Thus, we performed an RNA binding assay in which the 5′UTR of ADAM10 was labeled by 5‐bromo‐UTP (BrU).^[^ ^34^ ^]^ The in vitro synthesized BrU‐5′UTR recognized by BrU antibody‐beads was mixed with cytoplasmic proteins from SH‐SY5Y cells, and the resultant 5′UTR‐bound proteins were separated by electrophoresis for LC‐MS/MS analysis (Figure 4D). We found two sets of RBPs including 45 and 26 proteins in CTRL and KEN, respectively (Supplemental Table‐RBPs, and Figure S3, Supporting Information). Further protein‐protein interaction (PPI) analysis by using the online STRING software resulted in a complete network (Supplemental Figure S4); and a simplified diagram showed that SHMT2 interacted with canonical RBPs and ribosomal proteins (Figure 4E). The canonical RBP Y‐Box binding protein 1 (YBX1) interacted with the cold shock domain containing E1 (CSDE1),^[^ ^35^ ^]^ while the large and small subunits of ribosomal protein (RPLs/RPSs) interacted with each other, which formed connections with canonical RBPs. The interaction of SHMT2, along with YBX1 and CSDE1, with the 5′UTR was further verified by RNA‐pulldown assay (Figure 4F). These results indicated that SHMT2 was one of the key components of the RBP network targeting the 5′UTR, which was in line with a prior report demonstrating that SHMT2 is included in an atlas of mammalian RBPs.^[^ ^36^ ^]^ Further Western blotting experiments confirmed SHMT2 protein levels were significantly increased by KEN in a dose‐dependent manner (Figure 4G), and SHMT2 knockdown abolished KEN‐induced enhancement of ADAM10 expression (Figure 4H), indicating that SHMT2 mediated the effect of KEN on ADAM10 in vitro. It is reported that the small molecule Glycyrrhetinic acid (GA) directly binds to SHMT2 and suppresses mitochondrial energy metabolisms,^[^ ^37^ ^]^ whereas another compound Cosmosiin enhances ADAM10 protein level via the 5′UTR‐dependent mechanism by our prior report.^[^ ^16a^ ^]^ To prove the effect of KEN on ADAM10 and the 5′UTR, we assessed ADAM10 and SHMT2 protein levels in SH‐SY5Y cells treated with KEN (0.75 µM for 36 h), in the absence or presence of SHMT2 inhibitor GA (100 µM) or Cosmosiin (Cos, 5 µM). As shown in Figure 4I, GA treatment significantly reduced the protein level of ADAM10, and further blocked KEN‐induced augmentation of ADAM10 protein. Moreover, Cos incubation caused a significant elevation of ADAM10 protein level, and additional KEN did not cause further enhancement. These results indicated that small molecules interfering with SHMT2 or the 5′UTR abolished KEN‐induced regulation of ADAM10 translation.

### 2.5 SHMT2 Knockdown Attenuated the Effect of KEN on ADAM10 and Cognitive Function in APP/PS1 Mice

**Figure 5:**
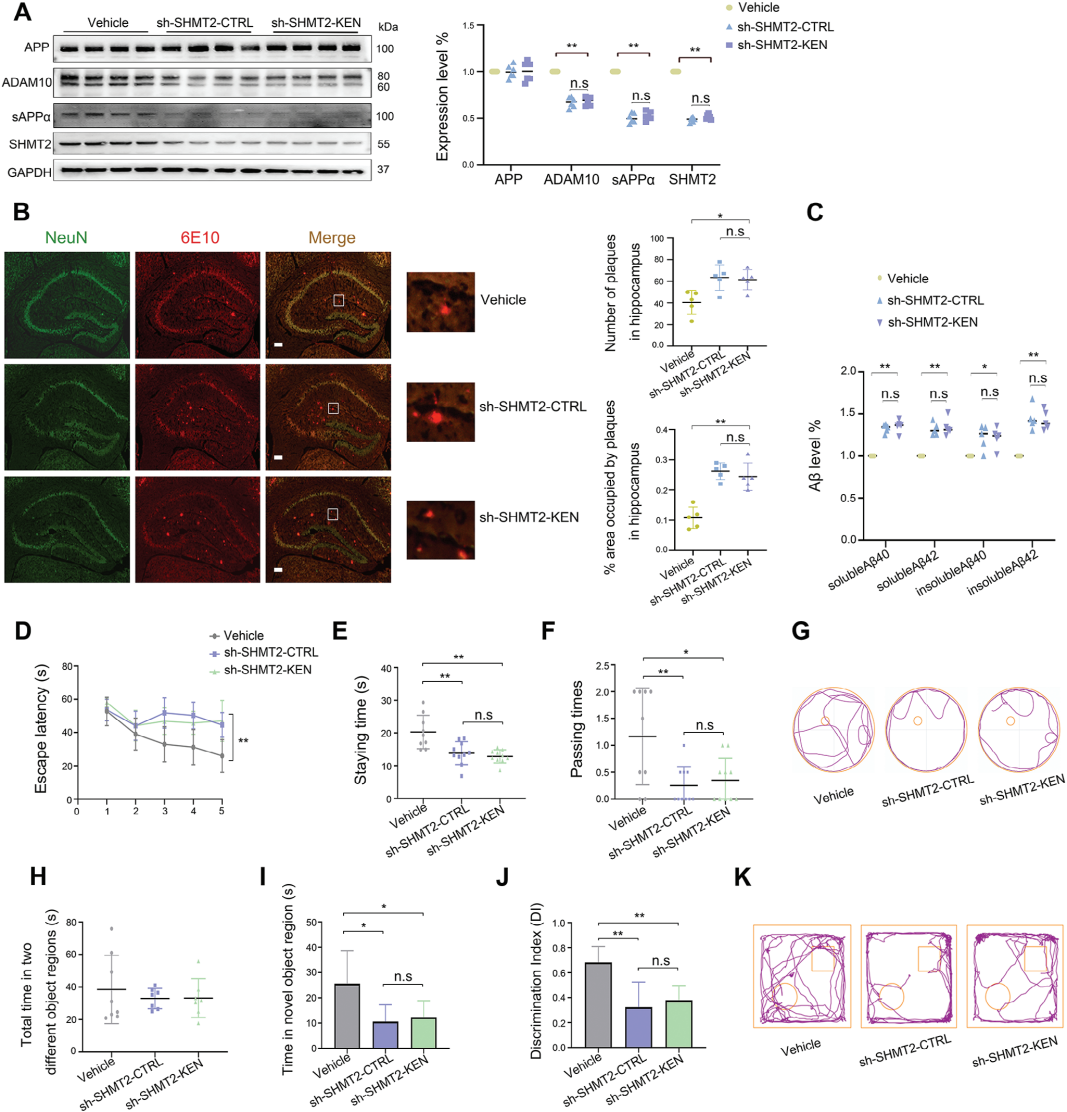
SHMT2 knockdown attenuates the effect of KEN on ADAM10 and cognitive function in APP/PS1 mice. A) Immunoblots (left) and quantification (right) of APP, ADAM10, sAPPα and SHMT2 in the hippocampus of APP/PS1 mice (male at 6‐month) injected with AAV vehicle (Vehicle), AAV‐SHMT2 shRNA in the absence (sh‐SHMT2‐CTRL) or presence of KEN (sh‐SHMT2‐KEN), *n* = 6 in each group. B) Immunofluorescence images (left) and quantifications (right) of Aβ load (6E10, red) in the hippocampus of APP/PS1 mice of three different groups. NeuN (green) is a marker of neuron, *n* = 6 in each, scale bar = 100 µm. C) Aβ1‐40/1‐42 levels measured by ELISA in the hippocampus of Vehicle, sh‐SHMT2‐CTRL and sh‐SHMT2‐KEN, respectively. D–G) Cognitive performances assessed by Morris water maze test, showing the escape latency D), staying times E) and passing times F), and the representative movement trajectories G) in different groups indicated, *n* = 9–10 in each. H–K) Novel object recognition tests show the total investigation time in two different object regions (H), the exploration time in the novel (the round one) object region I), the average discrimination index (DI, J), and the representative movement trajectories K) in APP/PS1 mice of three groups indicated, *n* = 7–8 in each. All values were normalized to vehicle control (CTRL) in each experiment. Data are presented as mean ± SEM from three or more independent experiments. n.s: no significant difference. ^*^ *p* < 0.05, ^**^ *p* < 0.01.

We next determined whether SHMT2 also mediated the effect of KEN on ADAM10 and amyloidogenesis in vivo. ADAM10 protein level in association with Aβ load and memory functions were assessed in APP/PS1 mice that were bilaterally injected in the hippocampus with viral AAV‐vector (Vehicle) or AAV‐shSHMT2 in the absence (AAV‐shSHMT2‐CTRL) and presence of KEN (AAV‐shSHMT2‐KEN). As shown in **Figure** **5**A, a significant reduction of SHMT2 protein was concomitantly accompanied by a significant decrease of ADAM10 and sAPPα levels; and KEN failed to cause an efficient enhancement of ADAM10/sAPPα when SHMT2 was silenced. Moreover, SHMT2 knockdown significantly increased the number and intensity of hippocampal Aβ, as well as Aβ40/42 levels, which were not altered by KEN (Figure 5B,C). Further behavioral testing revealed that the spatial and objective memories were significantly impaired by SHMT2 knockdown alone, whereas additional KEN failed to cause further behavioral alterations (Figure 5D–K). These results indicated that KEN‐induced regulation of ADAM10, amyloidogenesis, and cognitive function was mediated by SHMT2 in APP/PS1 mice.

### 2.6 SHMT2 Bound to Large Numbers of RNAs Critically Involved in AD and Cellular Functions of KEN

**Figure 6:**
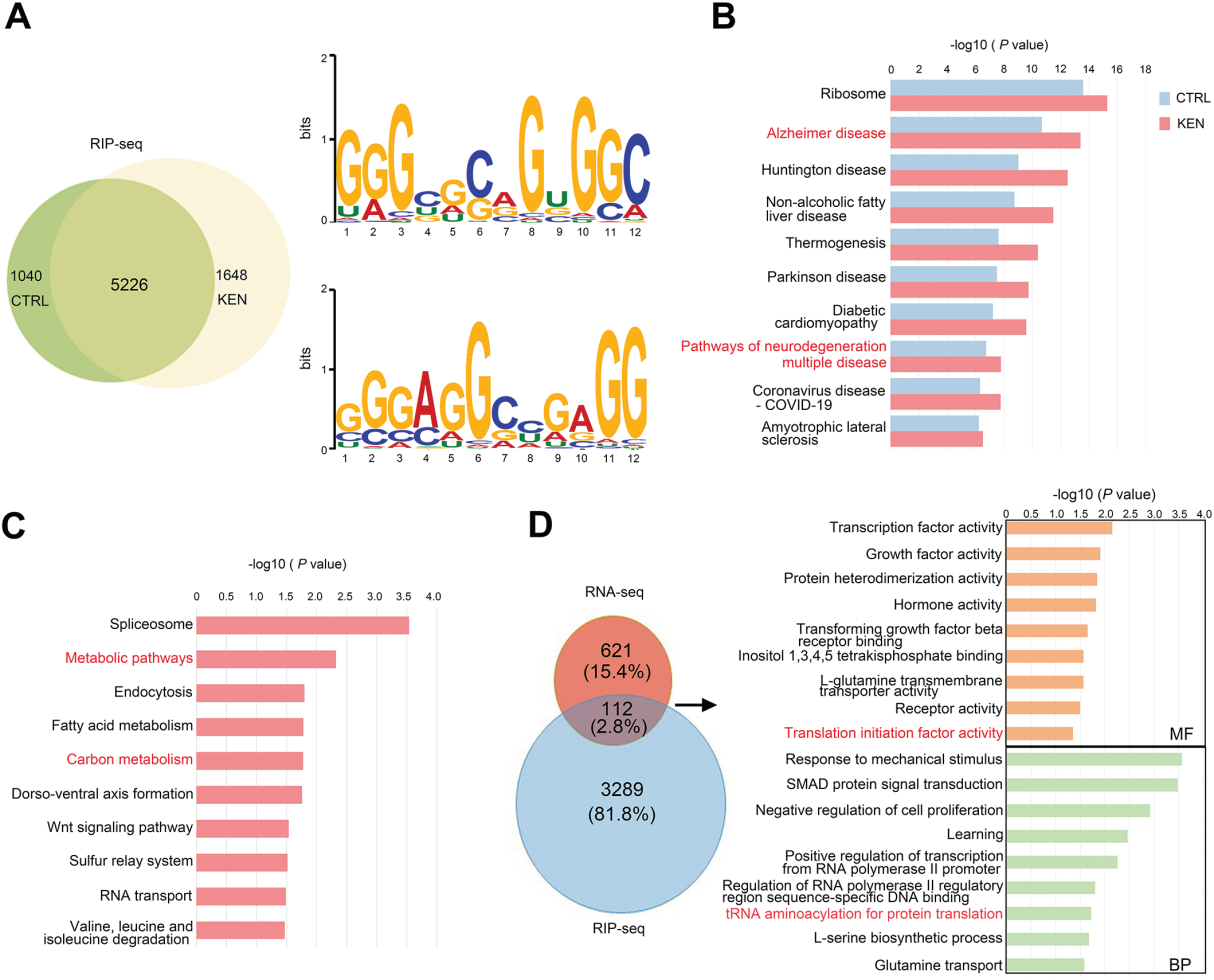
SHMT2‐targeted RNAs are involved in AD and the cellular function of KEN. A) Motif analysis of SHMT2‐targeted genes. Left, the Venn diagram shows SHMT2 recruits a total of 6266 and 6874 transcripts in control (CTRL) and KEN‐treated cells, respectively, in which 5226 genes are shared. Right, the most significant binding motifs recognized by SHMT2 are common in both CTRL and KEN, using MEME program. The horizontal axis denotes the base position in the corresponding motif, and the vertical axis shows the bit score with an E‐value of 4.9e‐004 (top) and 2.5e‐003 (bottom), respectively. B,C) KEGG analyses of SHMT2‐targeted RNAs show the most significantly enriched pathways, which are either shared by control (CTRL) and KEN (B), or specifically regulated by KEN relative to CTRL (C). D) A fraction of SHMT2‐targeted mRNAs affected by KEN are also included in KEN‐induced DEGs. Left, the Venn diagram shows 112 genes that are overlapped between SHMT2‐targeted mRNAs and the DEGs induced by KEN. Right, GO analysis of these overlapped genes.

The RNA‐binding property suggested that SHMT2 could regulate cellular functions through RNA processing in addition to its enzymatic activity. Thus, we performed RIP‐seq in SH‐SY5Y cells using an antibody against intrinsic SHMT2. As shown in **Figure** **6**A, SHMT2 recruited a total of 6266 and 6874 transcripts in control and KEN‐treated cells, respectively (Supplemental Table RIP‐Seq). Using the MEME program,^[^ ^38^ ^]^ motif analysis of 5226 RNAs that were commonly found in both groups revealed that SHMT2 preferentially interacted with GA‐ and GC‐rich motifs (Figure 6A). Importantly, KEGG pathways in both groups included ribosome, AD, and neurodegeneration of multiple diseases (Figure 6B). KEN specifically affected 3401 transcripts including those exclusively presented in either control or KEN, and altered by KEN in both groups; KEGG analysis revealed metabolic pathways and carbon metabolism that were consistent with SHMT2 function, whereas other pathways including RNA transport were also included (Figure 6C). As RNA‐protein interaction regulates RNA processing,^[^ ^39^ ^]^ we speculated that part of SHMT2‐targeted RNAs might contribute to KEN‐induced alteration of cellular functions. Taking advantage of DEGs data by KEN (Figure 4A,B), we assessed SHMT2‐targted mRNAs that were altered in KEN‐treated cells. Pooled analysis revealed that 112 genes were overlapped (Figure 6D), and GO analysis showed that transcription factor activity and importantly translation initiation factor activity were affected, indicating that a subset of SHMT2‐binding RNA abundance was particularly regulated by KEN. These results indicated that SHMT2 acted as RBP by preferentially interacting with GA/GC‐rich sequences, which were critically involved in AD and the cellular function of KEN.

### 2.7 SHMT2 Controlled the 5′UTR Activity Through Direct Binding to GAGGG Motif

**Figure 7:**
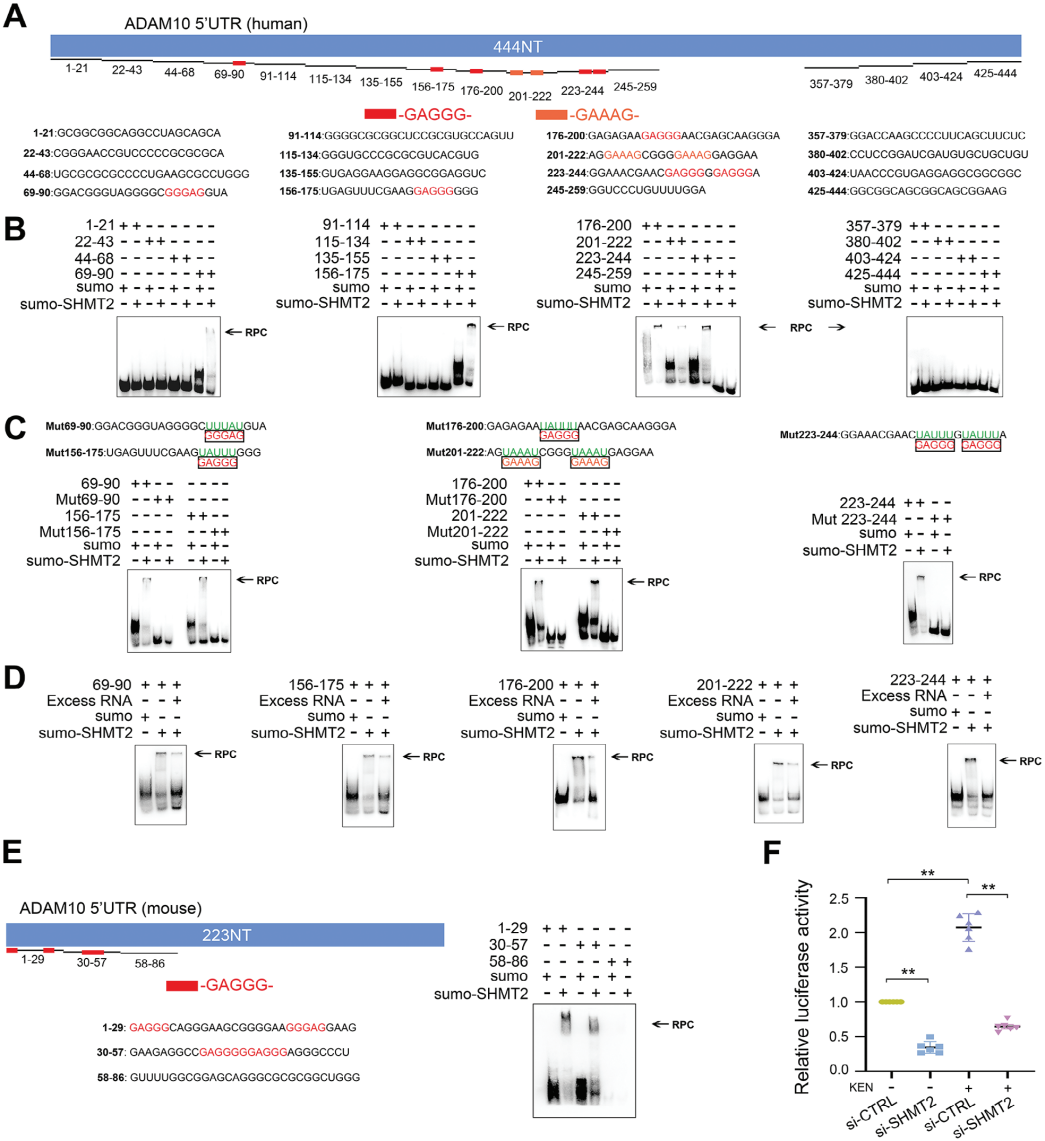
SHMT2 binds to GAGGG motif and controls the 5′UTR activity. A) Schematic diagram shows the ADAM10 5′UTR (Human) sequence that contains GAGGG or GAAAG at indicated positions (top). Different fragment sequences are numbered corresponding to positions in full‐length 5′UTR, in which GAGGG or GAAAG motif is marked red (bottom). Individual RNA sequence was labeled with biotin at the 5′ends. (B) RNA EMSA images show the binding of human SUMO‐tagged recombinant SHMT2 to different fragments of human 5′UTR. RNA‐protein complex (RPC) is only presented in fragments containing GAGGG or GAAAG motif. C) The numbered fragment sequences where GAGGG or GAAAG motif was mutated as indicated (top); and RNA EMSA images show that SHMT2 does not bind to the mutated fragments (bottom). (D) RNA EMSA images show human SHMT2 binding to 5′UTR in the presence of a 100‐fold excess of non‐labeled RNA probe that contains the same numbered sequence. E) The schematic diagram shows the murine ADAM10 5′UTR sequence that contains GAGGG; the numbered sequences with or without GAGGG are shown (top). Murine SUMO‐tagged recombinant SHMT2 (SUMO‐SHMT2) only binds to the numbered sequence that contains GAGGG as shown by RPC (bottom). SUMO: small ubiquitin‐like modifier. Each experiment was repeated for at least three times. F) Relative luciferase activity of the 5′UTR of human ADAM10 in HEK‐293 cells cotransfected with interfering RNA of CTRL (si‐CTRL) or SHMT2 (si‐SHMT2), in the presence or absence of KEN (1 µM for 36 h), *n* = 6. All values were normalized to vehicle control (CTRL) in each experiment. Data are presented as mean ± SEM from three or more independent experiments. n.s: no significant difference. ^*^ *p* < 0.05, ^s^ *p* < 0.01.

To validate that SHMT2 is directly bound to RNAs and exerted its function, we performed EMSA using different fragments of the 5′UTR (NM_0 01110) in ADAM10 mRNA.^[^ ^40^ ^]^ As shown in **Figure** **7**A, human full‐length 5′UTR contains multiple GAGGG/GAAAG sequences at indicated positions. The recombinant SHMT2 protein was tagged by a small ubiquitin‐like modifier (SUMO), which alone did not bind to any of these fragments (Figure 7B). SHMT2 only bound to GAGGG/GAAAG containing fragments that were numbered 69–90, 156–175, 176–200, 201–222, and 223–244, respectively. In contrast, the RNA‐protein complex (RPC) was absent where the RNA fragment did not include GAGGG/GAAAG motif (Figure 7B). Moreover, when GAGGG/GAAAG motif was mutated in the correspondingly numbered fragments, no RPC was found (Figure 7C). We further showed that in the presence of 100‐fold excessive nonlabeled RNA corresponding to the numbered fragment that bound to SHMT2, the RPC density was significantly reduced (Figure 7D). Because KEN also enhanced ADAM10 expression in murine cell lines and primary neurons (Figure 1), we then assessed SHMT2‐5′UTR binding of murine origin. As shown in Figure 7E, the 223nt‐long mouse 5′UTR (NM_0 07399) contains multiple GAGGG but not GAAAG motifs. The SUMO‐tagged murine SHMT2 only bound to fragments 1–29 and 30–57 that contained GAGGG, but not the fragment 58–86 that was without GAGGG; and SUMO alone did not bind to any of these fragments. To further confirm that SHMT2 controlled the 5′UTR function, we assessed the 5′UTR activity in HEK cells transiently with SHMT2 siRNA. As shown in Figure 7F, SHMT2 knockdown significantly reduced the luciferase activity of human 5′UTR, and significantly attenuated KEN‐induced augmentation. These results indicated that SHMT2 is directly bound to GAGGG motif of both human and murine origin, and regulated the 5′UTR activity.

### 2.8 GAGGG Mutation Affected Ribosomal Scanning by eIF2

**Figure 8:**
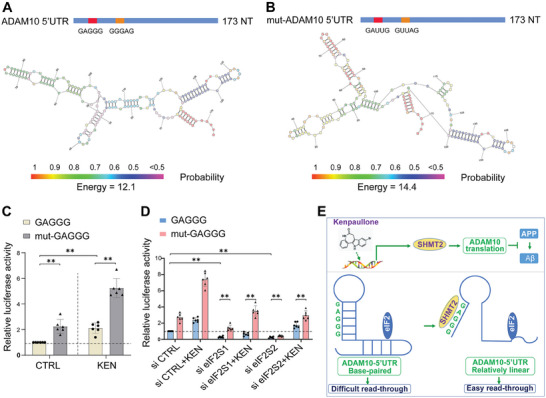
GAGGG mutation relieves the inhibition of the 5′UTR activity by eIF2 knockdown. (A&B) Predicted secondary structures of the 5′ UTR of human ADAM10 (NM_0 01110), in which GAGGG motif at 26 and 56 nt A) is mutated to GAUUG B). The colored bars (bottom) denote base paring probability, with the corresponding free energy values of the entire RNA structure. C) Relative luciferase activities of the 5′UTR that contain either GAGGG or GAUUG (mut‐GAGGG) in HEK293 cells in the absence (CTRL) or presence of KEN (1 µM for 36 h). (D) Relative luciferase activities of the 5′UTR with GAGGG or mut‐GAGGG in HEK293 cells transiently co‐transfected with interfering RNA of CTRL (si‐CTRL), eIF2S1 (si‐eIF2S1), or eIF2S2 (si‐eIF2S2), in the absence (CTRL) or presence of KEN (1 µM for 36 h), respectively. Data are presented as mean ± SEM. ^*^ *p* < 0.05, **p < 0.01 (ANOVA, *n* = 6). (E) A schematic model shows that KEN increases SHMT2 expression and the resulting ADAM10 translation, thus inhibiting Aβ generation from APP (top). In the 5′UTR of ADAM10, GAGGG motif functions as TIE by intrinsic base‐paring, and SHMT2 that directly binds to this motif might facilitate structural alteration of the 5′UTR, allowing an easy read‐through process of eIF2 complex, and the enhanced translation efficiency (bottom). All values were normalized to vehicle control (CTRL) in each experiment. Data are presented as mean ± SEM from three or more independent experiments. ^*^ *p* < 0.05, ^**^ *p* < 0.01.

The motif preference of SHMT2 suggested an important role of cis‐elements in regulating RNA function, especially the 5′UTR activity of ADAM10. To dissect the potential role of GAGGG motif in the 5′UTR function, wild‐type GAGGG located at 26 and 56 nt of the 5′UTR (ADAM10 5′UTR) was mutated to GAUUG (mut‐ADAM10 5′UTR) and cloned into luciferase reporter constructs. The corresponding secondary structures were predicted by an online resource http://rna.urmc.rochester.edu/RNAstructureWeb.^[^ ^41^ ^]^ As shown in **Figure** **8**A,B, a relatively well base‐pairing correlated with the 5′UTR structure containing GAGGG, whereas an altered and discontinued base‐paring was along with the different overall structure in mut‐ADAM10 5′UTR. In support, mut‐ADAM10 5′UTR showed higher free energy relative to control.^[^ ^42^ ^]^ Surprisingly, GAGGG mutation (mut‐GAGGG) led to a significantly enhanced luciferase activity of the 5′UTR in cells either without or with KEN treatment (Figure 8C), indicating that the GAGGG motif acted as a translation inhibitory element (TIE), which is reported to block translation of homeobox A9.^[^ ^43^ ^]^

Translation initiation is controlled by cis‐elements that work in concert with trans‐elements including eukaryotic initiation factors (eIFs).^[^ ^44^ ^]^ Interestingly, eIF2S1 (eIF2α) was identified as one of the interacting proteins in the 5′UTR of ADAM10 (Supplemental Figure S3, Supporting Information). To further determine whether this TIE/GAGGG motif might influence the read‐through process by eIF2S1 (eIF2α) and its partner eIF2S2 (eIF2β), we assessed the 5′UTR luciferase activity in cells transiently transfected with siRNA of eIF2S1/2. As shown in Figure 8D, knockdown of eIF2S1 or eIF2S2 alone, dramatically reduced the luciferase activity of GAGGG. However, irrespective of eIF2S1/2 knockdown, the luciferase activity of mut‐GAGGG remained significantly increased compared with that of GAGGG in cells with or without KEN treatment. These results indicated that mut‐GAGGG disinhibited the 5′UTR activity by eIF2 knockdown, supporting that an altered RNA structure might contribute to the enhanced ribosomal scanning.

Collectively, the presence of TIE/GAGGG motif is closely associated with RNA structure and translation efficiency. It is thus likely that SHMT2 binding to GAGGG motif could mimic the effect of GAGGG mutation by changing the intrinsically base‐paired 5′UTR into a relatively linear structure, which facilitates an easy read‐through by eIF2 complex and ADAM10 translation corresponding to the reduced amyloidogenesis (Figure 8E).

## 3 Discussion

The major findings of the present study include the following: 1) the therapeutic potential of KEN for AD is expanded by impeding amyloidogenesis, in addition to inhibiting CDKs/GSKs that are implicated in Tauopathy;^[^ ^29^, ^45^ ^]^ 2) the metabolic enzyme SHMT2 moonlights as the 5′UTR‐targeted RBP, and directly links OCM with amyloidogenesis through ADAM10 translation initiation; 3) GAGGG motif that acts as TIE highlights an important mechanism of SHMT2‐centered RNA processing in the pathophysiology of AD.

The present study reveals a novel role of KEN in ADAM10 translation, which is associated with a unique chemical structure but not inhibitory activity against CDKs/GSK‐3. The following evidence supports that KEN promotes ADAM10 expression through translation initiation: 1) ADAM10 mRNA is not altered; 2) translation inhibitors CHX and 4EGI‐1, but not transcription inhibitor ActD or protein degradation inhibitors CQ and MG132, abolish ADAM10 augmentation by KEN; 3) KEN fails to increase ADAM10 when the 5′UTR is lacking, and the luciferase activity of several fragments of the 5′UTR is enhanced by KEN. The function of the elevated ADAM10 is further demonstrated by the accompanying alteration of sAPPα and Aβ levels in human cells and the brain of APP/PS1 mice. Thus, the enhanced ADAM10 contributes to the rescue of cognitive deficits in KEN‐treated APP/PS1 mice, supporting that ADAM10 could serve as a therapeutic target for AD.^[^ ^46^ ^]^

Alteration of RBPs is a prominent feature in the brain of AD,^[^ ^35^, ^47^ ^]^ whereas the potential role of RBPs in amyloidogenesis remains unclear. In the present study, we have built a list of RBPs targeting the 5′UTR of ADAM10, thus providing a comprehensive source for further studying upstream signaling associated with ADAM10 and amyloidogenesis. Among these RBPs, only a subset of ribosomal proteins are included, highlighting that ribosomal heterogeneity is responsible for specific mRNA translation.^[^ ^48^ ^]^ Given that SHMT2 is a target gene of KEN and the 5′UTR‐interacting protein, the functional role of SHMT2 is further confirmed by that knockdown of SHMT2 reduces ADAM10 protein under basal condition and further prevents KEN‐induced enhancement of ADAM10 in human cells and the brain of APP/PS1 mice, which are with the concomitant improvement of amyloidogenesis and cognition.

Several metabolic enzymes “moonlight” as RBPs.^[^ ^49^ ^]^ It is reported that iron‐regulatory protein 1 binds mRNAs and regulates cellular functions.^[^ ^50^ ^]^ Interestingly, the cytosolic SHMT (SHMT1) is shown to regulate SHMT2 by binding to the 5′UTR.^[^ ^51^ ^]^ In our study, we provide evidence that SHMT2 binds to large numbers of RNAs that are associated with AD and multiple neurodegenerative diseases (Figure 6B), highlighting the important role of RNA processing in linking SHMT2 with the pathophysiology of AD. In addition to its enzymatic activity in OCM,^[^ ^6^, ^7^ ^]^ a fraction of the SHMT2‐targeted RNAs that are specific for KEN also involve metabolic pathways and OCM (Figure 6C), which are consistent with KEN‐induced DEGs (Figure 4A,B). The functional role of SHMT2‐RNA binding is further verified by that SHMT2 directly binds to GAGGG motif in the 5′UTR of ADAM10, and that knockdown of SHMT2 significantly inhibits the 5′UTR activity in the absence and presence of KEN. Thus, SHMT2‐RNA binding in association with GAGGG motif is critically involved in ADAM10 translation initiation, and to a broad‐spectrum, RNA processing related to the pathophysiology of AD.

TIE in the 5′UTR could fold into a specific RNA structure and block the read‐through of ribosomal proteins.^[^ ^43^, ^52^ ^]^ In the 5′UTR of ADAM10, the cis‐element G‐quadruplex inhibits translation by forming a stable secondary structure,^[^ ^53^ ^]^ supporting an important role of TIE in translational control.^[^ ^54^ ^]^ In our study, a comparison of the predicted structures of the 5′UTR shows an altered base‐paring and linearity, which correlates with the rather increased 5′UTR activity in mut‐GAGGG relative to GAGGG under basal conditions and in KEN‐treated cells. Similarly, an enhanced luciferase activity by mut‐GAGGG is also presented in cells transfected with siRNAs of eIF2S1/S2, indicating that GAGGG motif functions as TIE. Although the potential mechanisms are currently unclear, SHMT2 binding to this TIE might disinhibit ADAM10 translation through molecular interaction that alters the intrinsic base‐paring and the 5′UTR structure, in a similar way to GAGGG mutation.

A variety of eIFs cooperatively work with ribosomal proteins in regulating cap‐dependent and cap‐independent translation.^[^ ^44^, ^55^ ^]^ The secondary structure of the 5′UTR could form an internal ribosomal entry site, and recruit cap‐independent translation enhancers including ribosomal proteins and eIF4G and eIF3.^[^ ^56^ ^]^ In our study, eIF2S1 (eIF2α) is found in the list of the 5′UTR‐targeted RBPs, and silencing of eIF2S1/2 dramatically inhibits the 5′UTR‐luciferase activity, indicating that eIF2 directly regulates the 5′UTR activity. As several tRNA levels are also significantly increased by KEN (Supplemental Table‐DEGs), it could be possible that eIF2S1 and eIF2S2 (eIF2β) form a complex with the initiator tRNAs and guide an efficient read‐through in the initiation step.^[^ ^57^ ^]^ This mechanism could allow enhanced activity of the 5′UTR as a result of SHMT2‐RNA binding and the structural change of RNA.

We propose a model which KEN promotes ADAM10 translation by enhancing SHMT2 expression. Under basal conditions, GAGGG motif functions as TIE with intrinsic base‐paring in the 5′UTR, corresponding to a basal level of translation. In KEN‐treated cells, the increased SHMT2, through binding to GAGGG motif, primes an easy read‐through by eIF2 complex that is associated with an altered base‐paring and relatively linear structure of the 5′UTR, leading to an enhancement of ADAM10 translation initiation (Figure 8).

In summary, the present study shows that the metabolic enzyme SHMT2 also regulates RNA processing implicated in AD signaling pathways, and in particular mediates the effect of KEN on the 5′UTR activity of ADAM10. However, the multifaceted roles played by SHMT2 in the regulation of numerous biological pathways do not support the possibility of utilizing the association of KEN‐SHMT2 as a therapeutic approach to AD, and the reduced amyloidogenesis is just one of the multiple consequences mediated by SHMT2. Moreover, the improved cognitive function induced by KEN in animal models also involves a reduced Tauopathy that might be irrespective of SHMT2, as KEN is an inhibitor of GSK and CDKs that are known to phosphorylate Tau.^[^ ^58^ ^]^ Thus, the detailed function of SHMT2 in the pathophysiology of AD remains to be clarified in the future.

## 4 Experimental Section

### Cell Culture

Human neuroblastoma cell line SH‐SY5Y, and human embryonic kidney cell line HEK293 were purchased from the Type collection of the Chinese Academy of the Science (Shanghai, China), immortalized mouse hippocampal neuronal cell line HT22 was gifted from Dr. J Yang (Zunyi Medical University). HEK‐APP cells and SH‐SY5Y‐APP cells (HEK‐293 or SH‐SY5Y cells stably expressing full‐length human amyloid‐beta precursor protein 695) were generated as previously described.^[^ ^16^, ^26^ ^]^ Cells were maintained in the DMEM/F12 or DMEM medium (Gibco), supplemented with 10% fetal bovine serum (Biological Industries) and 1% penicillin/streptomycin (Thermo Fisher Scientific). Primary hippocampal neurons were extracted and cultured as described previously.^[^ ^16a^ ^]^

### Western Blotting

Proteins were extracted by using Minute Total Protein Extraction Kit for Animal Cultured Cells/Tissues (Invent Biotechnologies) or digested in RIPA buffer (Beyotime, Haimen, China) (0.5% sodium deoxycholate,1% Triton X‐100, 0.1% SDS, 1 mm EDTA, 150 mm NaCL and 50 mm Tris), supplemented with protease inhibitors (Roche, Indianapolis, IN, U.S.A.) and phosphatase inhibitors (Beyotime, Haimen, China), according to the manufacturer's instructions. Soluble extracellular proteins and insoluble material extraction were isolated as described previously.^[^ ^16b^ ^]^ Cell lysates were extracted using RIPA buffer (Beyotime, Haimen, China). sAPPα was extracted according to the protocol described previously.^[^ ^59^ ^]^ Protein concentration was measured by a BCA Protein Assay Kit (Dingguo, Beijing, China). Protein samples were loaded on 8%–12% SDS‐PAGE gels or 16.5% Tris‐tricine gels (for CTF detection) and were transferred to a PVDF membrane (Biorad, U.S.A.). Signals were captured by FX5 image analysis system (Vilber Lourmat) and analyzed by Quantity One software (Bio‐Rad).

### Immunofluorescence

Mice were sacrificed followed by perfused with ice‐cold phosphate buffer saline (PBS), the brains were isolated and post‐fixed in 4% paraformaldehyde (PFA), then transferred brains into 30% sucrose in PBS solution to make them dehydration until they were totally saturated. Put prepared brains in a freezing microtome (Leica, German) to obtain 15 µm sequential coronal sections, subsequently, the sections were moved onto the glass slides. After permeabilized with 0.3% Triton X‐100 in PBS and blocked with 10% goat serum (BOSTER Biological Technology, China) for 30 min at room temperature, the brain sections were collected to incubate with targeted antibodies according to the previously published procedures.^[^ ^60^ ^]^ When the in vitro cultured cells were used for immunofluorescence assays, cells on coverslips were fixed with 4% paraformaldehyde, permeabilized in 0.3% Triton X‐100, and blocked with 10% goat serum. Cells were then incubated with primary antibody of ADAM10 (Abcam) for 4 °C overnight, stained with goat anti‐rabbit secondary antibody Alexa Fluor 488 (Beyotime, China) for 60 min at room temperature. Then, coverslips were mounted with DAPI Fluoromount‐G (Southern Biotech, Birmingham, Alabama, USA). Images were captured by laser scanning confocal microscope (Leica TCS SP8 X, Germany) and quantified by Image‐J software (National Institutes of Health, USA).

### Cell Viability Assay

Cell viability assay was performed using Cell counting kit‐8 CCK‐8 (Beyotime, China). In brief, according to the manufacturer's instructions, SH‐SY5Y cells were seeded in a 96‐well plate and treated with different concentrations of Kenpaullone or equal volume DMSO for 36 h when cell density reached 70%–80% confluence. Optical density values were determined at 450 nm by using a Spectra Max 340 PC (Molecular Devices, Sunnyvale, CA, USA).

### Enzyme‐Linked Immunosorbent Assay (ELISA)

The levels of Aβ1‐40 or Aβ1‐42 were measured using an enzyme‐linked immunosorbent assay (ELISA) kit (Elabscience, Wuhan, China) according to the manufacturer's instructions.

### Small Interfering RNA and Transfection

The small interfering RNA (siRNA) for humans were purchased and synthesized from Shanghai GenePharma Co. Specific siRNAs sequence targeting human CDK5, GSK‐3β, YBX1, CSDE1, FDPS, SHMT2, eIF2S1 and eIF2S2 are listed in Table S1 (Supporting Information). Lipofectamine 2000 or 3000 Transfection Reagent (Thermo Fisher Scientific) was used for cellular transfection according to the manufacturer's instructions.

### Plasmid Construction and Luciferase Activity Assay

Human ADAM10 constructs containing or deleting the 5′UTR were kindly gifted from Dr. Sven Lammich (Ludwig‐Maximilians‐University, Germany). Human genomic DNA from cultured cells by Mammalian Genomic DNA Miniprep kit (Dinguo, Beijing, China) was used as a template for amplification of nucleotides included in pGL4.17‐ADAM10‐D, C, E1, E2, and F, respectively, using corresponding primer sequences (Table S2, Supporting Information ) described previously.^[^ ^33^ ^]^ PCR‐amplified fragments were subsequently cloned into the firefly luciferase reporter vector pGL4.17 (Promega, Madison, WI, USA). Luciferase activity was measured by a GloMax 96 microplate luminometer (Promega) in SH‐SY5Y cells transiently transfected with plasmids for 48 h, in the presence of 750 nm Kenpaullone for 36 h.

### RNA Extraction and Real‐Time Quantitative PCR (q‐PCR)

Total RNA from SH‐SY5Y cells was isolated by using RNAiso plus (Takara). cDNA was reverse transcribed using 5×HiScript II Select qRT Super Mix II (Vazyme, China) according to the manufacturer's instructions. Primers were synthesized by Sangon Biotech (Shanghai, China) and were listed in Supplemental Materials. The relative expression level of RNA was measured by qPCR system of AceQ qPCR SYBR Green Master Mix (Vazyme, China).

### RNA Sequencing Assay

SH‐SY5Y‐APP cells were treated with 750 nm Kenpaullone or an equal volume of DMSO for 36 h before the total RNA was isolated with RNAiso plus (Takara). Bioanalyzer 2100 system (Agilent) and Nanodrop spectrophotometer (Thermo Fisher Scientific) were used to identify the RNA integrity and quality. Each experiment was performed with three independent biological replicates. The sequencing library was constructed in accordance with the manufacturer's instructions. RNAseq assay was performed on the Illumina platform using an Illumine Novaseq 6000 instrument by Shanghai Applied Protein Technology (Shanghai, China). Per Kilo bases per Million reads (RPKM) values were calculated from each gene, to analyze the differentially expressed genes (*p* < 0.05, |log2 Fold change| > 0.5) between CTRL and KEN by using DESeq R package. Differentially expressed genes were used to do the GO analysis and KEGG pathway, in which P value < 0.05 was considered to be enriched. Differentially expressed genes (*p* < 0.05, |log2 Fold change| > 1) between CTRL and KEN by using DESeq R package were selected to obtain Co‐Expression analysis. Raw data were uploaded to the Sequence Read Archive (SRA) database (BioProject ID: PRJNA716891).

### RNA Binding Assay

RNA binding assay was performed according to the protocol of RiboTrap Kit (MBL International).^[^ ^34^ ^]^ Briefly, the 5′ UTR of ADAM10 mRNA was synthesized using Riboprobe System‐T7 (Promega). During the in vitro transcription process, the UTP was replaced by 5‐bromo‐UTP (BrU) using pcDNA3.1 plasmid with or without the 5′ UTR as a template. The synthesized BrU‐labeled 5′UTR was immunoprecipitated by anti‐BrU antibodies that were conjugated with protein G beads (Pierce). SH‐SY5Y cells were treated with 750 nm Kenpaullone or an equal volume of DMSO for 36 h. Cytoplasmic extracts were mixed with the 5′ UTR conjugated beads for 2 h and were washed and eluted before sampling. The separated proteins in SDS/PAGE were silver stained (Beyotime, China) and detected by LC‐MS/MS using a Q Exactive mass spectrometer (Thermo Fisher Scientific).

### RNA Immunoprecipitation and Sequencing (RIP‐seq)

RIP assays were performed with the Magna RIP RNA‐binding protein immunoprecipitation kit (Millipore, Billerica, MA, USA), according to the manufacturer's instructions. In brief, SH‐SY5Y cells were harvested in RIP lysis buffer, then incubated overnight at 4 °C with gentle rotation, with 7 µg SHMT2 antibody (Genetex) or an equal volume of control IgG that was conjugated to magnetic beads. Bioanalyzer 2100 system (Agilent) was used for RNA quality control. TruSeq Stranded RNA Sample Preparation Kit (Illumina) was used to construct the RNA library. High‐throughput sequencing was performed on IlluminaHiSeq 2500 by Seqhealth (Wuhan, China). Clean reads were normalized to calculate Per Kilo bases per Million reads (RPKM), the gene in which FPKM from IP compared to input was computed in a fold change > 2 and *p*‐value < 0.05 was chosen as significant enrichment. To analyze the GO analysis by using GOseq R package. To determine which gene is commonly bind but significantly altered in CTRL and KEN group, the genes with *p* < 0.05, |log2 Fold change| > 0.5 between 2 groups were selected to be analyzed. Motif identification was queried in MEME program (https://meme‐suite.org/meme/doc/meme.html). The search parameters were as follows: maximum width 12 amino acids, minimum motif width 4 amino acids, and other parameters were as defaults.

### RNA Electrophoretic Mobility Shift Assay (EMSA)

EMSA was performed using a LightShift Chemiluminescent RNA EMSA kit (Thermo Fisher Scientific). RNA probes were chemically synthetized by Sangon Biotech (Shanghai, China), which were labeled with biotin at the 5′ ends and purified by High‐Performance Liquid Chromatography (HPLC). Detailed RNA sequences corresponding to the human (NM_0 01110) and murine (NM_0 07399) 5′UTR were listed in Figure 7 and Tables S3 and S4. Recombinant SHMT2 of human (NP_0 05403.2) and mouse (NP_08 2506.1) origin was synthesized by GeneCreate Biological Engineering (Wuhan, China). According to the manufacturer's instructions, the binding reaction mixture included 10 pMol biotin‐labeled RNA probe, 2 µL 50% glycerol, 0.2 µL 10 mg mL^−1^ tRNA, 6 µg purified recombinant protein, 2 µL 10 X EMSA binding buffer and nuclease‐free water to a final volume of 20 µL. In the competitive reaction test, a 100‐fold excess of unlabeled RNA probes were added to the binding reactions. The mixture was incubated for 30 min at room temperature separated by electrophoresis in native PAGE then transferred to a positively charged Nylon membrane (Beyotime, China). After UV cross‐linking, HRP–conjugated streptavidin was used to detect RNA binding (Thermo Fisher Scientific).

### Mouse Models

APP/PS1 mice carrying Swedish APP and Presenilin one delta exon nine mutations (APPswe, PSEN1dE9) were purchased from the Jackson laboratory and bred in the Experimental Animal Center of Chongqing Medical University. All the experimental protocols were approved by the Ethics Committee of Chongqing Medical University following international standards. To assess the effect of kenpaullone (KEN) alone, APP/PS1 or wild‐type mice (12‐month‐old, female) with the same background identified by genotyping were i.p. injected with KEN (7 mg k^−1^g, every other day) for 2 months. For SHMT2 shRNA combined with KEN experiments, APP/PS1 mice (6‐month‐old, male) were randomly divided into three groups: bilateral hippocampus injection of adeno‐associated virus bearing control RNA, and SHMT2 shRNA without and with KEN (7 mg k^−1^g, every other day) for 2 months.

### Adeno‐Associated Virus (AAV)‐Mediated Transfection in the Hippocampus

SHMT2 shRNA experiment was conducted by using AAV serotype 2/9, which was designed to knockdown SHMT2 in vivo. AAVs were constructed by OBiO Technology (Shanghai, China), using shRNA: pAAV‐U6‐shSHMT2‐CMV‐EGFP‐WPRE, which titers are 3.05E + 12vg/ml, and pAAV‐U6‐ shNC2‐CMV‐EGFP‐WPRE to treat with mice in different groups. Murine shRNA sequences of SHMT2 were as follows: sh‐SHMT2‐1: CTTCGAGTCTATGCCCTATAA; sh‐SHMT2‐2: ACTGGCAAAGAGATCCCTTAT and sh‐SHMT2‐3: CCTTTCAAGTACGCGGATGTT. The sequence of sh‐SHMT2‐2 exhibited the most effective knockdown result and was chosen for the next experiments. Mice were anesthetized with sodium pentobarbital and carefully moved in a stereotaxic instrument (RWD Life Science). Using glass microsyringe to collect 0.5 microliters of virus particles for each site and bilaterally inject them into CA1 and DG area of the hippocampus.^[^ ^61^ ^]^

### Immunohistochemistry

Brain sections were incubated with anti‐β‐Amyloid antibody 6E10 (Covance) overnight at 4 °C, followed by rapid treatment of DAB (Zhongshan Golden Bridge) and counterstained with Hematoxylin for nuclei.^[^ ^16b^ ^]^ The total number of 6E10 positive plaques in each section was quantified by ImageJ software and the average number of plaques per section was calculated (8–11 sections/mouse).

### Morris Water Maze and Contextual Fear Conditioning

The Morris water maze test included four platform trials per day for five consecutive days and a probe trial on the sixth day. Swimming activities including latency and distance were recorded by a video and analyzed by image analyzing software (ANY‐maze; Stoelting).^[^ ^62^ ^]^ Data were analyzed by two‐way ANOVA with Tukey's post hoc test. In the contextual and cued fear conditioning test, a 3‐day paradigm was used. Behavioral activity was recorded by video camera and freezing data were measured using FreezeScan software.^[^ ^63^ ^]^

### Novel Object Recognition Test

Two phases were included in the test: the familiarization phase and the testing phase (1 h after phase 1). Mice were observed in a rectangular cage (22 cm height × 44 cm length × 22 cm width). In the familiarization phase, mice were exposed to two identical objects (≈15 cm height × 4 cm width × 4 cm length) for 5 min, then removed from the experimental field allowing for a rest in the same environment for 1 h. In the testing phase, one of the objects was replaced by another one with a different shape, the remaining steps were the same as in phase 1. All behaviors and track of mice were video‐recorded.

### Statistical Analysis

All data were presented as mean ± standard error of the mean (SEM) and performed at least in biological triplicate unless otherwise stated, number of samples for test (N) was indicated in the figure legends individually. GraphPad Prism (version 9.0) or SPSS (version 25.0) software was used for statistical analysis. Statistical significance was computed using unpaired two‐tailed Student's t‐test or Mann‐Whitney *U*‐test, one‐way ANOVA (Dunnett's multiple comparison test or Turkey's multiple comparison test), and two‐way ANOVA where applied. In the case that data did not meet the assumption of homogeneity of variances, the Kruskal‐Wallis test was employed. *p* ≤ 0.05 was set as the threshold of significance.

## Conflict of Interest

The authors declare no conflict of interest.

## Author Contributions

G.‐J.C. designed the study. L.S. performed the experiments and analyzed the data. Q.‐L.P., G.‐F.Z., S.‐W.L., B.‐L.Z., P.‐J.L., X.‐T.H., J.‐S.Z., Y.L., Q.‐X.W., B.L., J.C., Y.T., J.T., X.‐J.X., X.‐Y.X., X.‐J.D. provided assistance with the research. G.‐J.C. and L.S. wrote the manuscript.

## Supporting information

Supporting Information

Supporting Information

Supporting Information

Supporting Information

## Data Availability

The data that support the findings of this study are available on request from the corresponding author. The data are not publicly available due to privacy or ethical restrictions.
