# Supplementary material for: SHMT2 Mediates Small‐Molecule‐Induced Alleviation of Alzheimer Pathology Via the 5′UTR‐dependent ADAM10 Translation Initiation
Source: Adv Sci (Weinh). 2024 Jan 6;11(11):2305260. doi: 10.1002/advs.202305260 (PMC10953581; doi:10.1002/advs.202305260)
Supplement: Supplementary file 1 — Supporting Information [file ADVS-11-2305260-s001.pdf]

ADVANCED SCIENCE
Open Access

Supporting Information

for *Adv. Sci.*, DOI 10.1002/advs.202305260

SHMT2 Mediates Small-Molecule-Induced Alleviation of Alzheimer Pathology Via the 5′UTR-dependent ADAM10 Translation Initiation

*Li Song, Qiu-Ling Pan, Gui-Feng Zhou, Sheng-Wei Liu, Bing-Lin Zhu, Pei-Jia Lin, Xiao-Tong Hu, Jing-Si Zha, Yan Long, Biao Luo, Jian Chen, Ying Tang, Jing Tang, Xiao-Jiao Xiang, Xiao-Yong Xie, Xiao-Juan Deng and Guo-Jun Chen**

Supplementary information for

SHMT2 mediates small molecule-induced alleviation of Alzheimer pathology via the 5'UTR-dependent ADAM10 translation initiation

Materials

Antibodies	SOURCE	IDENTIFIER
ADAM10	Abcam	Cat# ab124695; RRID: AB_10972023
ADAM10	Abcam	Cat# ab1997; RRID: AB_302747
BACE1	Abcam	Cat# ab2077; RRID: AB_302817
CDK5	Proteintech	Cat#10430-1-AP; RRID: AB_2078859
GSK-3β	Proteintech	Cat#22104-1-AP; RRID:AB_2878997
Anti-Amyloid Precursor Protein, C-Terminal antibody	Sigma	Cat# A8717; RRID: AB_258409
Anti-β-Amyloid,17-24 Antibody	Biolegend	Cat# SIG-39220; RRID: AB_662812
Anti-β-Amyloid,1-16 Antibody	Biolegend	Cat# SIG-39300; RRID: AB_662803
Anti-Human sAPP alpha (2B3)	IBL	Cat# 11088; RRID: AB_494690
GAPDH	Proteintech	Cat# 60004-1-Ig; RRID: AB_2107436
Anti-Tau (phospho S396)	Abcam	Cat# ab109390; RRID: AB_10860822
Anti-Tau (phospho S262)	Abcam	Cat# ab131354; RRID: AB_11156689
SHMT2	Genetex	Cat# GTX125939; RRID: AB_11174916
SHMT2	Abcam	Cat# ab88664; RRID: AB_2042908
CSDE1	Abcam	Cat# ab201688
YBX1	Abcam	Cat# ab12148; RRID: AB_2219278

NrCAM	Proteintech	Cat# 21608-1-AP;<br><br>RRID: AB_10859786
β-actin	Proteintech	Cat# 66009-1-Ig; RRID: AB_2687938
eIF2S1	Abmart	Cat#PA1376
eIF2S2	Abcam	Cat#ab184549
Goat anti-mouse IgG (H+L),<br><br>HRP conjugate	Proteintech	Cat# SA00001-1; RRID: AB_2722565
Goat Anti-Rabbit IgG(H+L),<br><br>HRP conjugate	Proteintech	Cat# SA00001-2; RRID: AB_2722564
**Chemicals, Peptides,**<br><br>**and Recombinant Proteins**	**SOURCE**	**IDENTIFIER**
Kenpaullone	Sigma-Aldrich	K3888
Kenpaullone	MedChemExpress	HY-12302
Alsterpaullone	Apexbio	B7855
AT7519	MedChemExpress	HY-50940
Dimethyl sulfoxide	Solarbio	D8371
Actinomycin D	MedChemExpress	HY-17559
Cycloheximide	MedChemExpress	HY-12320
Chloroquine diphosphate	MedChemExpress	HY-17589
MG132	MedChemExpress	HY-13259
4EGI-1	MedChemExpress	HY-19831
Glycyrrhetinic acid	MedChemExpress	HY-N0180
Cosmosiin	MedChemExpress	HY-N0578

DMEM	Gibco	8120345
DMEM/F12	Gibco	8120331
B-27 Supplement (50X), serum free	Gibco	17504044
Neurobasal	Gibco	21103049
L-Glutamine 200mM	Gibco	A2916801
Trypsin	Gibco	25200056
Fetal bovine serum	Biological Industries	04-121-1A
Penicillin/Streptomycin	Gibco	15140122
EDTA-free Protease Inhibitor Cocktail	Roche	4693159001
Phosphatase inhibitor cocktail D, 50X	Beyotime	P1096
Lipofectamine 2000 Transfection Reagent	ThermoFisher Scientific	11668019
Lipofectamine 3000 Transfection Reagent	ThermoFisher Scientific	L3000015
RNAiso plus	Takara	9109
Recombinant (SHMT2)	ImmunoClone	IC8217
**Cortical Commercial Assays**	**SOURCE**	**IDENTIFIER**
BCA assay kit	Beyotime	P0012S
Cell Counting Kit-8	Beyotime	C0037
Fast Silver Stain Kit	Beyotime	P0017S
5×HiScript II Select qRT Super Mix II	Vazyme	R233-01-AC
AceQ qPCR SYBR Green Master Mix	Vazyme	Q111-02
LightShift Chemiluminescent RNA EMSA kit	Thermo Fisher Scientific	20158

RiboTrap Kit	MBL International	RN1011/RN1012
Human Aβ 1-40 enzyme-linked immunosorbent assay kit	Elabscience	E-EL-HO542c
Human Aβ 1-42 enzyme-linked immunosorbent assay kit	Elabscience	E-EL-HO543c
EZ-Magna RIP RNA-binding protein	Millipore	17-701
Steady -Glo Luciferase assay	Promega	E2510
Riboprobe® System - T7	Promega	P1440
Deposited Data		
The RNA sequence data	This paper	SRA: PRJNA716891
**Experimental Models: Cell Lines**	**SOURCE**	**IDENTIFIER**
Human: HEK-293	ATCC	CRL-1573
Human: SH-SY5Y	ATCC	CRL-2266
Mouse：HT22	Shanghai Zhong Qiao Xin Zhou Biotechnology	ZQ0476
**Experimental Models: Organisms/Strains**	**SOURCE**	**IDENTIFIER**
Mouse：C57BL/6J (WT)	Jackson Laboratory	Cat#000664
mouse：Tg (APPswe,PSEN1dE9) 85Dbo	Jackson Laboratory	Cat#34832-JAX
**Oligonucleotides**	**SOURCE**	**IDENTIFIER**
qPCR: Human ADAM10 forward	This paper	ATGGGAGGTCAGTATGGGAATC
qPCR: Human ADAM10 reverse	This paper	ACTGCTCTTTTGGCACGCT
qPCR: Human GAPDH forward	This paper	CAGGAGGCATTGCTGATGAT
qPCR: Human GAPDH reverse	This paper	GAAGGCTGGGGCTCATTT

siRNA target sequence: see Table S1	Hu et al., 2017	N/A
PCR primers for gene cloning: see Table S2	This paper	N/A
RNA probes used in EMSA: see Table S3 and S4	This paper	N/A
**Recombinant DNA**	**SOURCE**	**IDENTIFIER**
Plasmid: ADAM10 (+5'UTR)	Dr. Sven Lammich,Ludwig-Maximilians-University, Germany	N/A
Plasmid: ADAM10 (+5'UTR)	Dr. Sven Lammich,Ludwig-Maximilians-University, Germany	N/A
**Software and Algorithms**	**SOURCE**	**IDENTIFIER**
GraphPad Prism V9	GraphPad Software	https://www.graphpad.com/
ImageJ	National Institutes of Health	https://imagej.nih.gov/ij/
SPSS	IBM Corp	https://www.ibm.com/cn-zh/products/spss-statistics

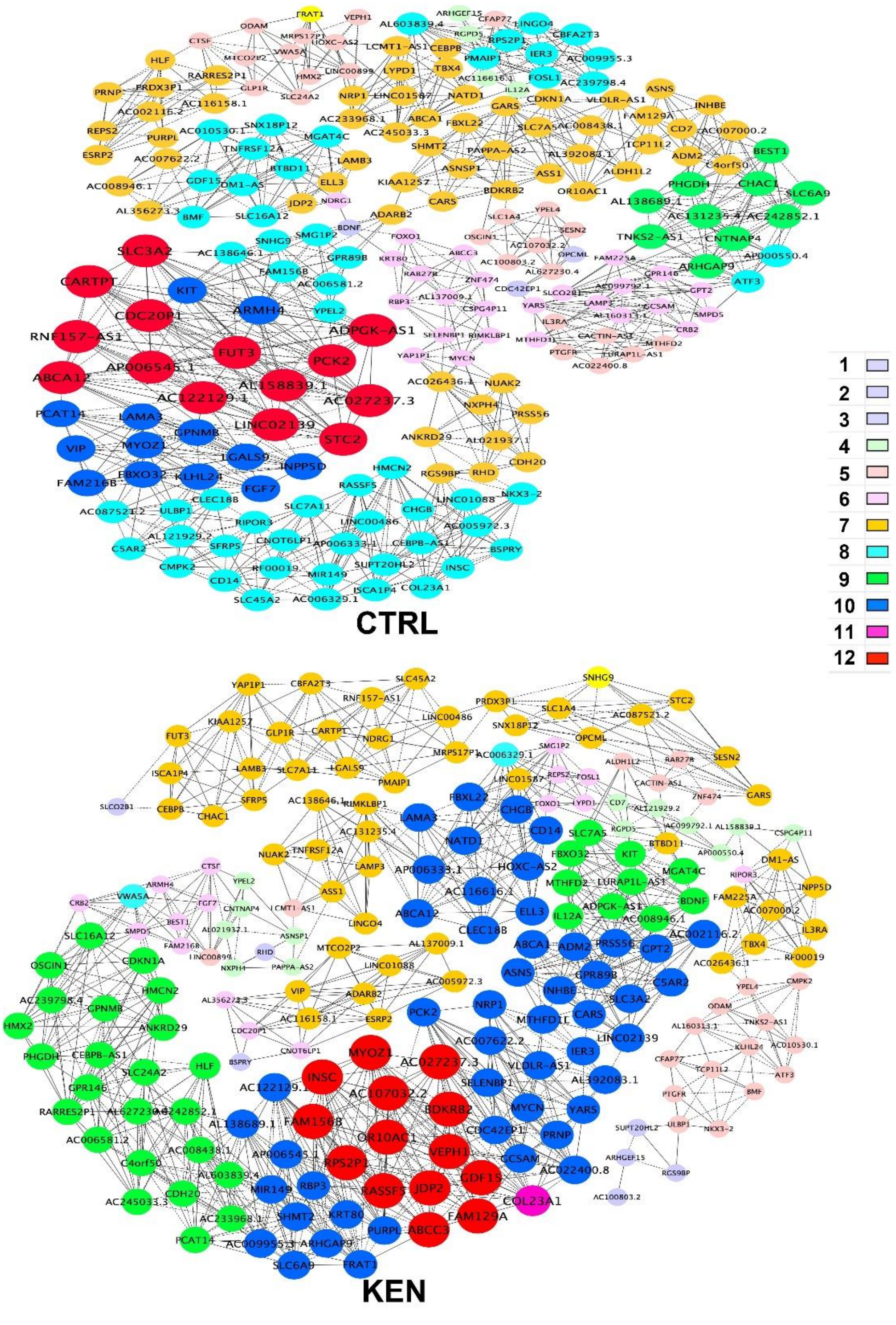

Figure S1. Co-Expression Analysis. Gene co-expression networks were built to find the relations among genes, in SH-SHY5Y-APP cell in the absence (CTRL) or presence of kenpaullone (KEN), respectively. （|log2 Fold change| > 1, $P < 0.5$). Genes in normalized expression values were selected according to significant GO-terms, pathway-terms and differentially expressed mRNA. To study some properties of the networks, K cores ranging from 1 to 12 with different colors were introduced as a method of simplifying graph topology

analysis [1]. For a specific gene, the alteration of K-core that reflects the complexity of gene association can be influenced by KEN. SHMT2 for example, is shown in yellow (K core = 7) in the top panel (CTRL), and in blue (K core = 10) in the bottom (KEN), with different interacting partners sharing the same colors.

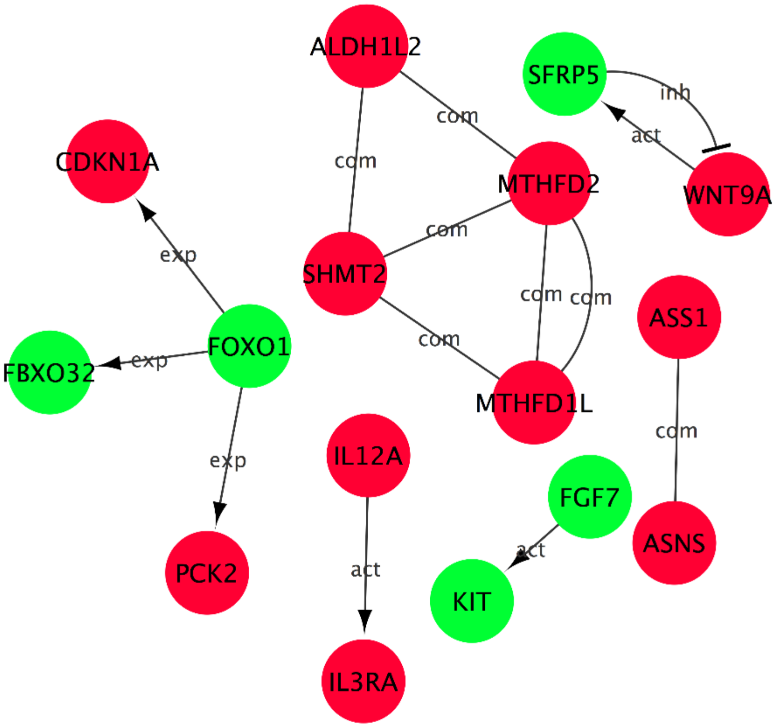

Figure S2. Gene-Act-Net. The relationship network of up/down-regulated genes in SH-SY5Y-APP cells in response to KEN was built according to KEGG database（|log2 Fold change| > 1, P < 0.5)..

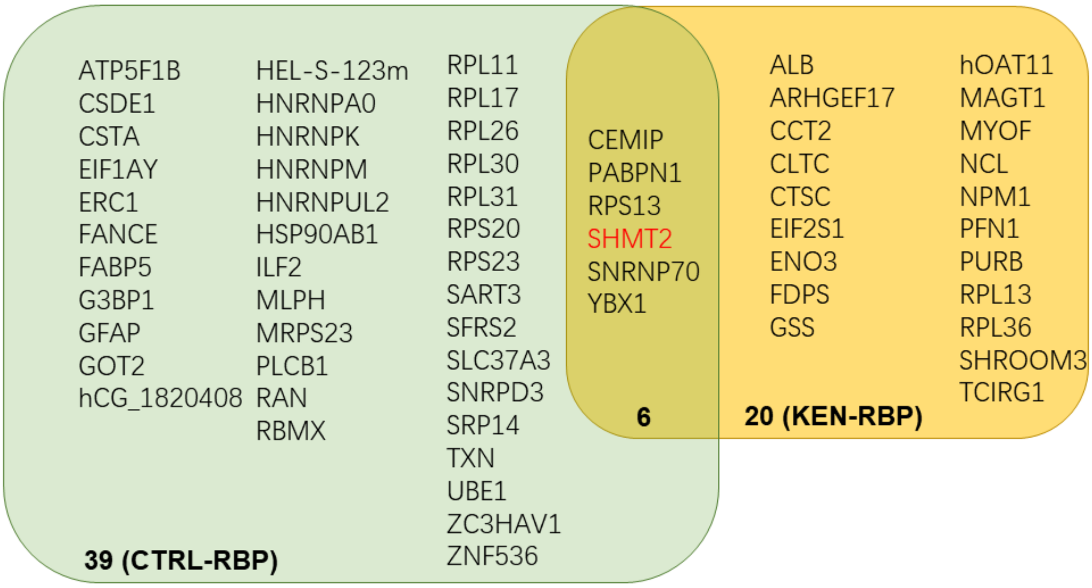

Figure S3. RBPs that target the 5'UTR of ADAM10 mRNA. Schematic diagram of the 5'UTR-interacting RBPs identified in SH-SY5Y cells in the presence or absence of KEN (0.75 μM for 36 h). A total of 45 and 26 proteins in CTRL and KEN, respectively, are listed, whereas 6 of them, including SHMT2, are found in both groups.

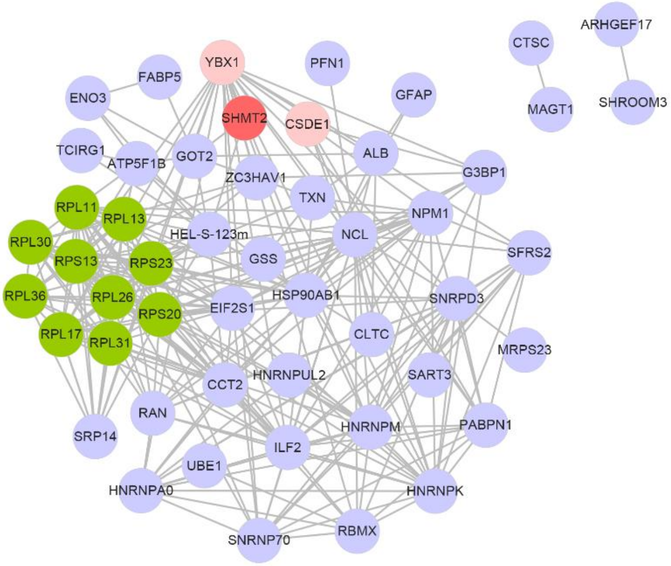

Figure S4. Interaction network of RBPs in response to KEN. According to STRING database, the predicted protein-protein interaction network was built using proteins identified by LC-MS/MS through RNA binding assay targeting the 5'UTR of ADAM10. Two sets of proteins were collected in cells in the absence and presence of the small molecule KEN, respectively. SHMT2 interconnects with canonical RBP- and ribosomal-centered proteins.

Table S1. siRNA sequence

Genes	siRNA Oligo
CDK5	5'-GCCAGACTATAAGCCCTAT-3'
GSK-3β	5'-GGACTATGTTCCGGAAACA-3'
SHMT2	5'-GGAGAGTTGTGGACTTTAT-3'
eIF2S1	5'-CTGGAATACAACAACATTGAA-3'
eIF2S2	5'-GTGCGAAACTTGTCATTCT-3'

Table S2. Primer sequence for fragments of ADAM10 plasmid

Gene	Primer sequence (5'-3')	Restriction site
ADAM10-C	CGGGGTACCGCGGCGGCAGGCCTAGC<br>CCGCTCGAGTCCTCACGGGTTAACAGCAGCACAT	KpnI<br>XhoI
ADAM10-D	CGGGGTACCAGCTCTCCGCCGGCGGAC<br>CCGCTCGAGTCCTCACGGGTTAACAGCAGCACAT	KpnI<br>XhoI
ADAM10-E1	CGGGGTACCAGCTCTCCGCCGGCG<br>CCGCTCGAGTCCTTCCTCACCACGTGACG	KpnI<br>XhoI
ADAM10-E2	CGGGGTACCGCGGCGGCAGGCCTA<br>CCGCTCGAGTCCTTCCTCACCACGTGACG	KpnI<br>XhoI

ADAM10-F	CTGGCCTAACTGGCCGGTACCAGGCGGAGGTCTGAGTTT<br><br>GCCAGATCTTGATATCCTCGAG	KpnI<br><br>XhoI

Table S3. RNA probes used in EMSA (human sequences of the 5'UTR of ADAM10)

Probe	sequence
1-21	GCGGCGGCAGGCCUAGCAGCA
22-43	CGGGAACCGUCCCCCGCGCGCA
44-68	UGCGCGCGCCCCUGAAGCGCCUGGG
69-90	GGACGGGUAGGGGCGGGAGGUA
91-114	GGGGCGCGGCUCCGCGUGCCAGUU
115-134	GGGUGCCCGCGCGUCACGUG
135-155	GUGAGGAAGGAGGCGGAGGUC
156-175	UGAGUUUCGAAGGAGGGGGG
176-200	GAGAGAAGAGGGAACGAGCAAGGGA
201-222	AGGAAAGCGGGGAAAGGAGGAA
223-244	GGAAACGAACGAGGGGGAGGGA
245-259	GGUCCCUGUUUUGGA
357-379	GGACCAAGCCCCUUCAGCUUCUC
380-402	CCUCCGGAUCGAUGUGCUGCUGU
403-424	UAACCCGUGAGGAGGCGGCGGC
425-444	GGCGGCAGCGGCAGCGGAAG
MUT69-90	GGACGGGUAGGGGCUUUAUGUA
MUT156-175	UGAGUUUCGAAGUAUUUGGG

MUT176-200	GAGAGAAUAUUUAACGAGCAAGGGA
MUT201-222	AGUAAAUCGGGUAAAUGAGGAA
MUT223-244	GGAAACGAACUAUUUGUAUUUA

Table S4. RNA probes used in EMSA (murine sequences of the 5'UTR of ADAM10)

Probe	sequence
1-29	GAGGGCAGGGAAGCGGGGAAGGGAGGAAG
30-57	GAAGAGGCCGAGGGGGAGGGAGGGCCCU
58-86	GUUUUGGCGGAGCAGGGCGCGCGGCUGGG

Table S5. The 5'UTR sequences used for luciferase assays.

Gene	sequence
ADAM10 5'UTR	GTTGCCGGCCCCTGAAGTGGAGCGAGAGGGAGGTGCT<br>TCGCCGTTTCTCCTGCCAGGGGAGGTCCCGGCTTCCCG<br>TGGAGGCTCCGGACCAAGCCCCTTCAGCTTCTCCCTCC<br>GGATCGATGTGCTGCTGTTAACCCGTGAGGAGGCGGC<br>GGCGGCGGCAGCGGCAGCGGAAG
Mutated ADAM10 5'UTR	GTTGCCGGCCCCTGAAGTGGAGCGAGATTGAGGTGCT<br>TCGCCGTTTCTCCTGCCAGGTTAGGTCCCGGCTTCCCG<br>TGGAGGCTCCGGACCAAGCCCCTTCAGCTTCTCCCTCC<br>GGATCGATGTGCTGCTGTTAACCCGTGAGGAGGCGGC<br>GGCGGCGGCAGCGGCAGCGGAAG
	ACAAGTCTTTCCGCCTCCCCAGCCCGCCCGGGAGCTG<br>CGAGCCGCGAGCTGGATTATGGTGGCCTGAGCAGCCA<br>ACGCAGCCGCAGGAGCCCGGAGCCCTTGCCCCTGCCC<br>GCGCCGCCGCCCGCCGGGGGGACCAGGGAAGCCGCC<br>ACCGGCCCGCCATGCCCGCCCCTCCCAGCCCCGCCGG<br>GAGCCCGCGCCCGCTGCCCAGGCTGGCCGCCGCCGTG

BACE1 5’UTR	CCGATGTAGCGGGCTCCGGATCCCAGCCTCTCCCCTG CTCCCGTGCTCTGCGGATCTCCCCTGACCGCTCTCCAC AGCCCGGACCCGGGGGCTGGCCCAGGGCCCTGCAGG CCCTGGCGTCCTGATGCCCCCAAGCTCCCTCTCCTGAG AAGCCACCAGCACCACCCAGACTTGGGGGCAGGCGC CAGGGACGGACGTGGGCCAGTGCGAGCCCAGAGGGC CCGAAGGCCGGGGCCCACC

Data file S1. Supplemental Table-DEGs (differentially expressed genes). (Excel file).

Data file S2. Supplemental Table-RBPs (RNA binding proteins). (Excel file).

Data file S3. Supplemental Table-RIP-seq (SHMT2 targeted RNAs). (Excel file).

[1] E. Ravasz, A. L. Somera, D. A. Mongru, Z. N. Oltvai, A. L. Barabasi, *Science* **2002**, 297, 1551.
